# Adipose Tissue Macrophages as Initiators of Exacerbated Periodontitis in Estrogen‐Deficient Environments via the Amplifier Extracellular Vesicles

**DOI:** 10.1002/advs.202506121

**Published:** 2025-07-12

**Authors:** Danfeng Li, Tian Yang, Yuqian Li, Xinwei Lyu, Cheng Hu, Jiayin Yan, Jiali Tan

**Affiliations:** ^1^ Hospital of Stomatology Sun Yat‐sen University Guangzhou 510055 China; ^2^ Guangdong Provincial Key Laboratory of Stomatology Guangzhou 510080 China; ^3^ Guanghua School of Stomatology Sun Yat‐sen University Guangzhou 510080 China

**Keywords:** estrogen deficiency, extracellular vesicles, macrophages, periodontitis, therapy

## Abstract

Periodontitis in menopausal women tends to exacerbate, potentially resulting in tooth loss and increased risk of systemic diseases. The prior research demonstrates that pro‐inflammatory changes in macrophages under estrogen deficiency exacerbate periodontitis. However, the primary environmental factors contributing to the alteration of monocyte‐macrophages remain unknown. Recent studies, including animal models and clinical trials, have found a correlation between accumulation of visceral adipose tissue (VAT) and periodontitis progression in postmenopausal women. In estrogen‐deficient mice, macrophages in VAT show a pro‐inflammatory state. Removing VAT alleviates periodontitis in OVX mice. DNA methylation sequencing shows increased methylation in macrophages, especially *Jazf1* hypermethylation, inhibiting its expression and promoting inflammation. Subsequently, small extracellular vesicles (sEVs) derived from pro‐inflammatory macrophages further intensify M1‐like polarization in resting macrophages, carrying inflammatory microRNAs like miR‐30e‐5p. Overall, this study proposes a novel perspective: periodontal pathogens act as initial triggers for inflammation, while chronic systemic inflammation, worsened by estrogen deficiency, is the main factor that exacerbates periodontitis. Pro‐inflammatory macrophages in VAT release sEVs, which activate resting macrophages into a pro‐inflammatory state upon encountering periodontal pathogens, resulting in persistent inflammation. Addressing the root causes of this dysregulation can lead to new therapeutic strategies for periodontitis and systemic inflammatory conditions, particularly in postmenopausal women.

## Graphical abstract

Chronic systemic inflammation, worsened by estrogen deficiency, underlies and exacerbates periodontitis. Proinflammatory macrophages within visceral adipose tissue are hyperactivated due to hyper‐DNA methylation, releasing small extracellular vesicles (sEVs) similar to those of their parent cells. When periodontal pathogens invade, quiescent macrophages at the site are activated into a proinflammatory state by endocytosing circulating sEVs, causing persistent inflammation.

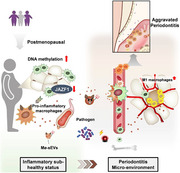

## 1 Introduction

Periodontitis constitutes a significant public health concern with profound implications for both the economy and mortality rates. This condition not only leads to tooth loss and compromised oral function but also contributes to the development of chronic ailments such as cardiovascular diseases, diabetes, hypertension, and cancer. According to the latest study published in the Journal of Clinical Periodontology in 2023, there is a notable prevalence of periodontitis. The study estimated that ≈62% of adults were affected by periodontitis between the years 2011 and 2020, with a significant ratio of 23.6% experiencing severe forms of the condition.^[^ ^1^ ^]^

Although bacterial plaque is acknowledged as the primary etiological factor in periodontitis, the host response to pathogenic stimulation is crucial in determining the disease's outcome. Consequently, systemic factors can also influence the progression of periodontitis. Previous research indicates a correlation between estrogen deficiency and the aggravated periodontitis, as it has been observed to contribute to an inflammatory status.^[^ ^2^ ^]^ The aged tendency of population has led to the emergence of menopause as an inevitable issue, prompting the use of complementary hormone therapy. However, this therapy is associated with side effects such as tumorigenesis. Accordingly, preventing periodontitis in postmenopausal women becomes a burning problem. Addressing this issue, our prior research identified that macrophages within periodontal tissues exhibit pro‐inflammatory alterations and impede the osteogenic differentiation of mesenchymal stem cells in the context of estrogen deficiency, thereby exacerbating periodontitis.^[^ ^3^ ^]^ Nonetheless, we consider that periodontal macrophages predominantly originate from monocytes in the bloodstream following activation by local pathogenic bacteria. This raises questions regarding the primary environmental triggers responsible for their pro‐inflammatory transformation.

In prior research, it was observed that the majority of ovariectomized (OVX) mice exhibited a pronounced phenotype characterized by the accumulation of visceral adipose tissue. Subsequent investigations indicated a correlation, to a certain degree, between the accumulation of abdominal fat tissue in postmenopausal women and the progression of periodontitis. Additionally, it was discovered that macrophages within the adipose tissue of OVX mice exhibited a pro‐inflammatory phenotype, and their periodontitis symptoms were mitigated following adipotomy. Consequently, we are interested in exploring the potential, yet unexplored, connection between adipose tissue and periodontal macrophages. Recent studies have further demonstrated an increase in visceral fat accumulation in mice subsequent to ovariectomy (OVX).^[^ ^4^ ^]^ It also suggests that the accumulation of abdominal fat is a significant characteristic of postmenopausal women.^[^ ^5^ ^]^ Additionally, it has been reported that postmenopausal women with obesity suffer from exacerbated periodontitis,^[^ ^6^ ^]^ but the underlying mechanisms remain unknown.

Adipose tissue is an active endocrine organ that secretes a variety of cytokines and molecules involved in immune regulation.^[^ ^7^, ^8^ ^]^ Depending on the distribution, adipose tissue can be categorized into subcutaneous adipose tissue (SAT) and visceral adipose tissue (VAT).^[^ ^9^ ^]^ The immune cells in visceral adipose tissue play a crucial role in regulating the homeostasis and inflammation levels of adipose tissue during pathological conditions, as well as in modulating their quantity and functions.^[^ ^10^ ^]^

Among the various immune cells, macrophages are particularly abundant in adipose tissue. Adipose tissue macrophages play a crucial role in modulating systemic cellular functions.^[^ ^11^ ^]^ On one hand, it is believed that adipose tissue serves as an additional reservoir for macrophages originating from the bone marrow, where undifferentiated monocytes infiltrate and differentiate into mature macrophages, potentially contributing to an increased macrophage population that might play greater roles in systemic inflammation.^[^ ^12^ ^]^ In healthy adipose tissue, the proportion of macrophages is typically less than 10% of the total cell population. On the contrary, in pathological circumstances, this ratio can increase significantly, reaching up to 50% due to the accumulation of circular mononuclear macrophages in adipose tissue.^[^ ^13^, ^14^ ^]^ Macrophages within visceral adipose tissue predominantly originate from the infiltration of monocyte‐macrophages derived from peripheral blood.^[^ ^15^ ^]^ Mature macrophages expressing F4/80 are more inclined to sustain an inflammatory phenotype. Researchers have demonstrated that visceral fat harbors a greater number of infiltrating macrophages and exhibits elevated levels of inflammatory cytokine expression relative to subcutaneous fat.^[^ ^16^ ^]^ The interaction between these macrophages and adipocytes exerts a prolonged influence on the body through metabolic processes and their dysfunction is closely linked to metabolic diseases.^[^ ^17^, ^18^, ^19^ ^]^ Notably, the polarization phenotypes of these macrophages play a crucial role in determining their diverse immune response functions. For instance, the M1 polarization of macrophages promotes inflammation, whereas the M2 polarization exerts anti‐inflammatory effects.^[^ ^20^, ^21^ ^]^

On the other hand, the substantial blood flow and active secretory functions of macrophages in adipose tissue confer a more pronounced systemic impact through secretion compared to macrophages located in bone marrow.^[^ ^22^ ^]^ However, the mechanisms by which these macrophages influence distal immune responses remain inadequately understood.

Among the secretions of VAT, a range of cytokines and molecules, including tumor necrosis factor (TNF), Interleukin‐6, and acute phase proteins, have the potential to induce a state of high oxidation. Although the underlying biological mechanism remains unclear, existing theories propose that alterations in the immune inflammatory profile of obese individuals might impact the immune response to periodontitis.^[^ ^8^, ^23^ ^]^ For the past few years, extracellular vesicles have emerged as stable vehicles for long‐range communication, exerting notable impacts on immune regulation.^[^ ^24^, ^25^, ^26^, ^27^ ^]^ Specifically, small extracellular vesicles (sEVs), measuring ≈30 – 200 nm in diameter, have been recognized as crucial intercellular regulators due to their ability to transfer bioactive molecules such as DNA, mRNA, non‐coding RNA, and proteins.^[^ ^3^, ^27^, ^28^ ^]^

Consequently, this study seeks to elucidate the primary changes occurring in macrophages under conditions of estrogen deficiency and their impact on pro‐inflammatory regulation. Furthermore, we intend to investigate the influence of the small extracellular vesicle (sEV) pathway, derived from pro‐inflammatory macrophages, on inflammation and the exacerbation of periodontitis. This will involve identifying key inflammatory molecules present in sEVs and elucidating their underlying mechanisms. In general, our study is expected to provide new a strategy for the treatment of not only periodontitis, but also other systemic inflammatory diseases in postmenopausal women.

## 2 Results

### 2.1 Severity of Periodontitis and Abdominal Obesity was Aggravated in Postmenopausal Women

**Figure 1:**
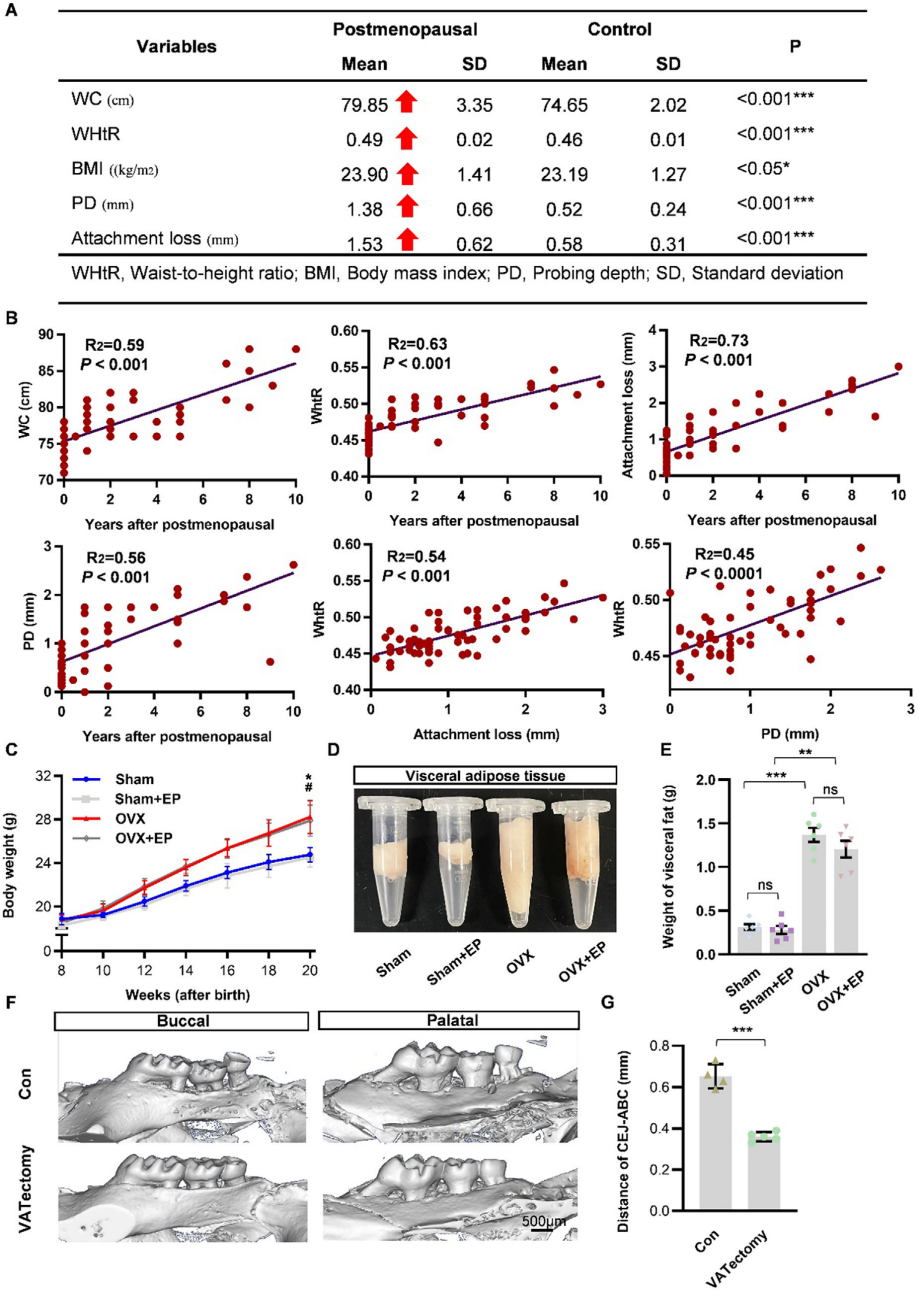
Periodontitis and abdominal obesity aggravated after estrogen deficiency. A) Comparison of anthropometric and biological parameters between premenopausal and postmenopausal women. B) Correlation between WC, WHtR, AL, PD and years after postmenopausal, respectively. And the correlation between AL, PD and WHtR. C) The body weight of C57BL/6 mice increased after OVX operation. Data represent mean ± SD. *P < 0.05 (OVX group compared with Sham group), #*P* < 0.05 (OVX+EP group compared with Sham+EP group). D,E) Visceral fat in the abdominal cavity accumulated after the OVX operation. Data are shown as mean ± SD, with n = 6 in each group, ns: not significant, **P < 0.01, ***P < 0.001 (One‐way ANOVA). F) Representative 3D reconstruction images of maxillae harvested 4 weeks after periodontitis induction from OVX+EP group (n = 4) or OVX+EP with VATectomy group (n = 5). G) The average linear distance from CEJ‐ABC of maxillary molars on both the buccal and palatal sides for each group. ***P <0.001 (Student's t test).

To explore the relationship between periodontitis and abdominal obesity in postmenopausal women. We investigated the periodontal probing depth (PD), attachment loss (AL), waist circumference (WC) and waist‐to‐height ratio (WHtR) of premenopausal and postmenopausal women (**Figure** **1**A). The results showed that the waist circumference (79.85 ± 3.35 cm) and waist‐to‐height ratio (0.49 ± 0.02) of postmenopausal women were significantly higher than those of pre‐menopausal women (74.65 ± 2.02 cm, *P* <0.001) and waist‐to‐height ratio (0.46 ± 0.01, *P* <0.001). In addition, probing depth (1.38 ± 0.66 mm) and attachment loss (1.53 ± 0.62 mm) in postmenopausal women were also aggravated in premenopausal women (PD, 0.52 ± 0.24 mm, *P* <0.001; AL, 0.58 ± 0.31 mm, *P* <0.001). The correlation between menopause age and WC, WHtR, AL, PD was determined by linear regression correlation analysis. The results showed that with increasing age after menopause, there was a positive correlation with WC, WHtR, AL and PD (Figure 1B). Furthermore, the link between abdominal obesity and periodontitis severity was verified. Importantly, WHtR was strongly and significantly associated with attachment loss (R^2^ = 0.54, *P* <0.001) and probing depth (R^2^ = 0.45, *P* <0.0001).

To further investigate the role of macrophages in exacerbated periodontitis under conditions of estrogen deficiency, we developed an experimental periodontitis (EP) model and simulated estrogen deficiency through ovariectomy (OVX). Subsequently, we randomly allocated the mice into four groups: Sham, OVX, Sham + EP, and OVX + EP. In the OVX mice with periodontitis, we observed an increase in body weight (Figure 1C) and visceral fat accumulation (Figure 1D,E). Our previous studies have demonstrated that periodontitis is exacerbated in OVX mice following periodontitis induction,^[^ ^3^ ^]^ and we recently observed significant accumulation of abdominal adipose tissue. Notably, periodontitis‐related bone loss was reduced in OVX mice following the removal of abdominal fat (Figure 1F,G), suggesting that estrogen deficiency facilitates abdominal fat accumulation, which is potentially associated with the progression of periodontitis.

### 2.2 M1‐Polarization of Macrophages was Enhanced in Periodontal Tissue and Visceral Adipose Tissue of OVX Mice with Periodontitis

**Figure 2:**
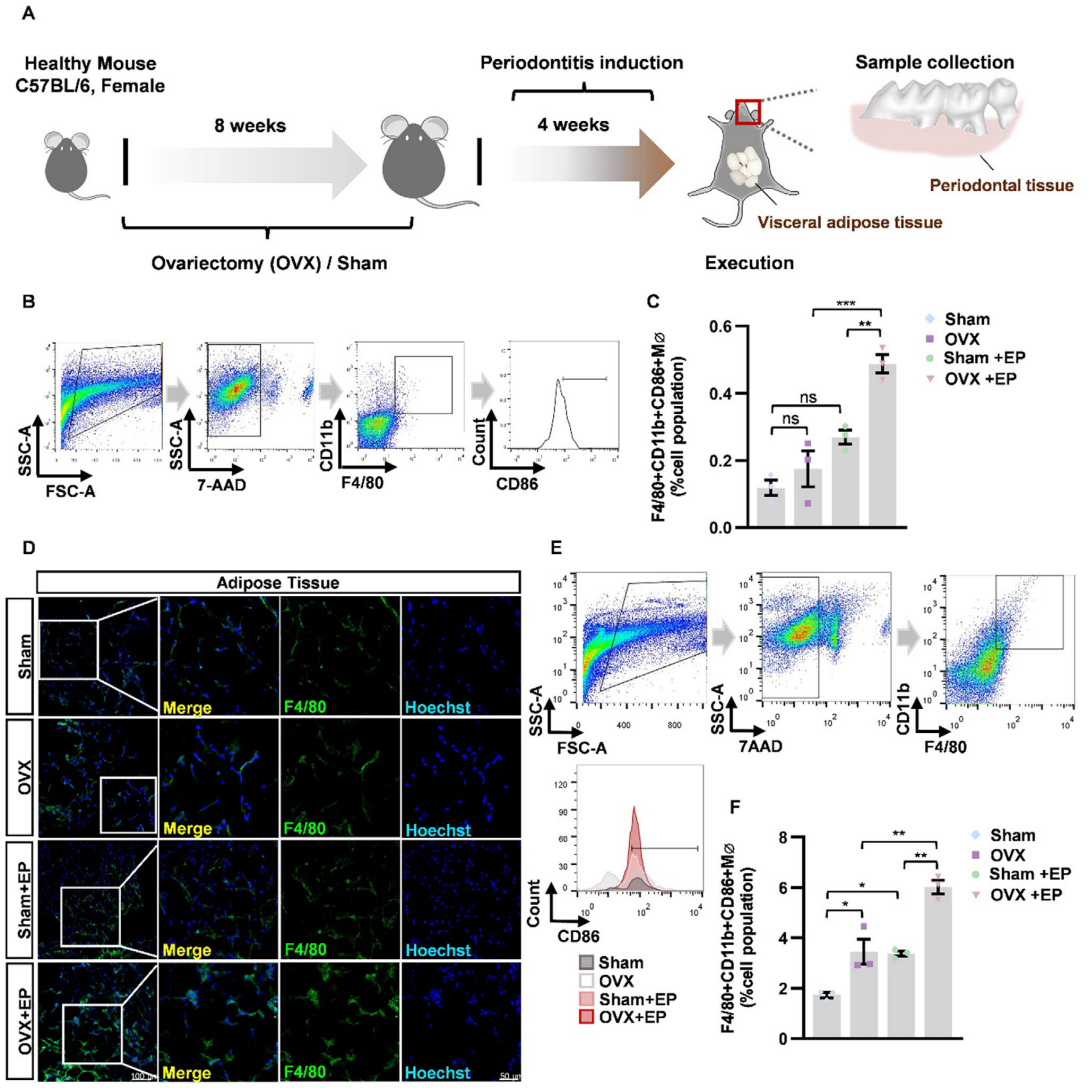
Enhanced M1 polarization in periodontal tissue and adipose tissue in OVX mice with periodontitis. A) A schematic overview of establishing a combined OVX mouse model for periodontitis. B,C) Representative live cell gates and scatter plots from Sham, Sham+EP, OVX and OVX+EP periodontal tissues identifying CD11b+ F4/80+ cells, further subdivided by low or high CD86 expression (percentage of total live cells), and corresponding group data. D) Representative confocal images of F4/80 immunofluorescence in abdominal adipose tissues from Sham, Sham+EP, OVX and OVX+EP mice. E F) Representative live cell gates and scatter plots from Sham, Sham+EP, OVX and OVX+EP adipose tissues identifying CD11b+F4/80+ cells, further subdivided by low or high CD86 expression (percentage of total live cells), and corresponding group data. Data represent mean ± SEM. *P <0.05, **P <0.01, ***P <0.001, ns: not significant. (One‐way ANOVA).

As macrophages have been reported as an important source of inflammation in adipose tissues, we collected the periodontal tissue of mice from Sham, OVX, Sham + EP, and OVX + EP groups (**Figure** **2**A), observing a significant increase of F4/80+ CD11b+ CD86+ macrophages in periodontal tissue, indicating enhanced M1‐polarized macrophages (Figure 2B,C). Given that the accumulation of abdominal adipose tissue is also a key feature of postmenopausal women, we obtained visceral adipose tissue of the above groups and conducted immunofluorescence staining and flow cytometry to detect the state of macrophages under different conditions. As data shown, there were numerous macrophages infiltrated in the visceral adipose tissue (Figure 2D). Consistent with the above data, the proportion of M1 macrophages in the adipose tissue increased in OVX mice, especially after periodontitis modeling (Figure 2E,F). Overall, the findings point to a possible connection between dysfunctional macrophages in the periodontium and those in visceral adipose tissue.

### 2.3 Macrophages were Critical for the Aggravated Periodontitis in OVX Mice

**Figure 3:**
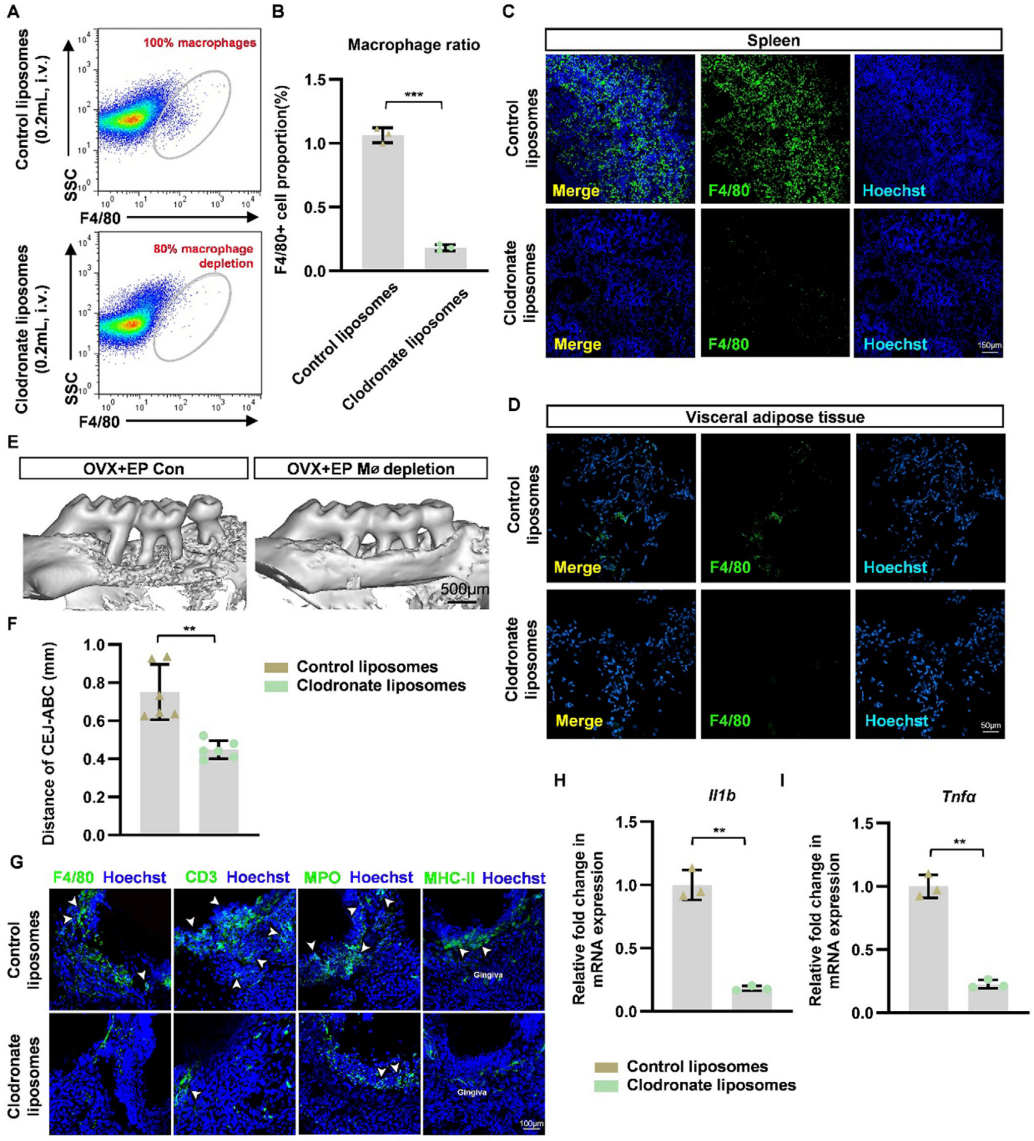
Macrophage depletion alleviated periodontitis in OVX mice. A) Gating strategy and representative flow cytometric plots of F4/80 expressing macrophages from the spleens of control‐liposome‐injected and clodronate‐liposome‐injected mice. B) Percentage of F4/80+ macrophages from the spleen of the two groups above. C) Representative immunofluorescence of the spleen after 24 h of injection, stained for F4/80 (green) and Hoechst (blue). D) Representative immunofluorescence of the visceral adipose tissue after 24 h of injection, stained for F4/80 (green) and Hoechst (blue). E) Reconstructed 3D images of maxillae harvested 4 weeks after periodontitis induction. F) The average linear distance from CEJ‐ABC of maxillary molars on both the buccal and palatal sides for each group (n = 6). G) F4/80, CD3, MPO and MHC‐II were used to monitor macrophages, T cells, neutrophils and MHCII‐positive antigen‐presenting cells, respectively, in the periodontal tissues of each group. The arrows indicate infiltrating positive cells. H,I) The mRNA levels of *Il1b* and *Tnfα* in periodontal tissues were determined by qPCR analysis. Results are shown as a fold change in expression with respect to the baseline of the control group. Data represent mean ± SD. * *P* <0.05, **P <0.01, ***P <0.001 (Student's t test).

To explore the role of macrophages in periodontitis, macrophages were cleared from mice through clodronate liposomes. According to F4/80 staining by fluorescence activated cell sorting (FACS) and immunofluorescence, more than 80% of macrophages in the spleen were cleared after 24 h of clodronate injection, demonstrating a successful depletion of macrophages (**Figure** **3**A–C). Furthermore, immunofluoresence of F4/80 in visceral adipose tissue also verified the removal of macrophages (Figure 3D). As data shown, the elimination of macrophages reduced alveolar bone loss (Figure 3E,F) and inflammatory‐cell infiltration in periodontal tissue in OVX mice with periodontitis (Figure 3G). Furthermore, qPCR analysis of the mRNA expression level of inflammatory genes *Il1b* and *Tnfα* also revealed alleviated inflammation in periodontal tissue after macrophage depletion (Figure 3H,I). Collectively, the data demonstrates the critical role of macrophages in aggravated periodontitis under estrogen deficiency.

### 2.4 DNA Methylation in Macrophages Alters under Estrogen Deficiency

**Figure 4:**
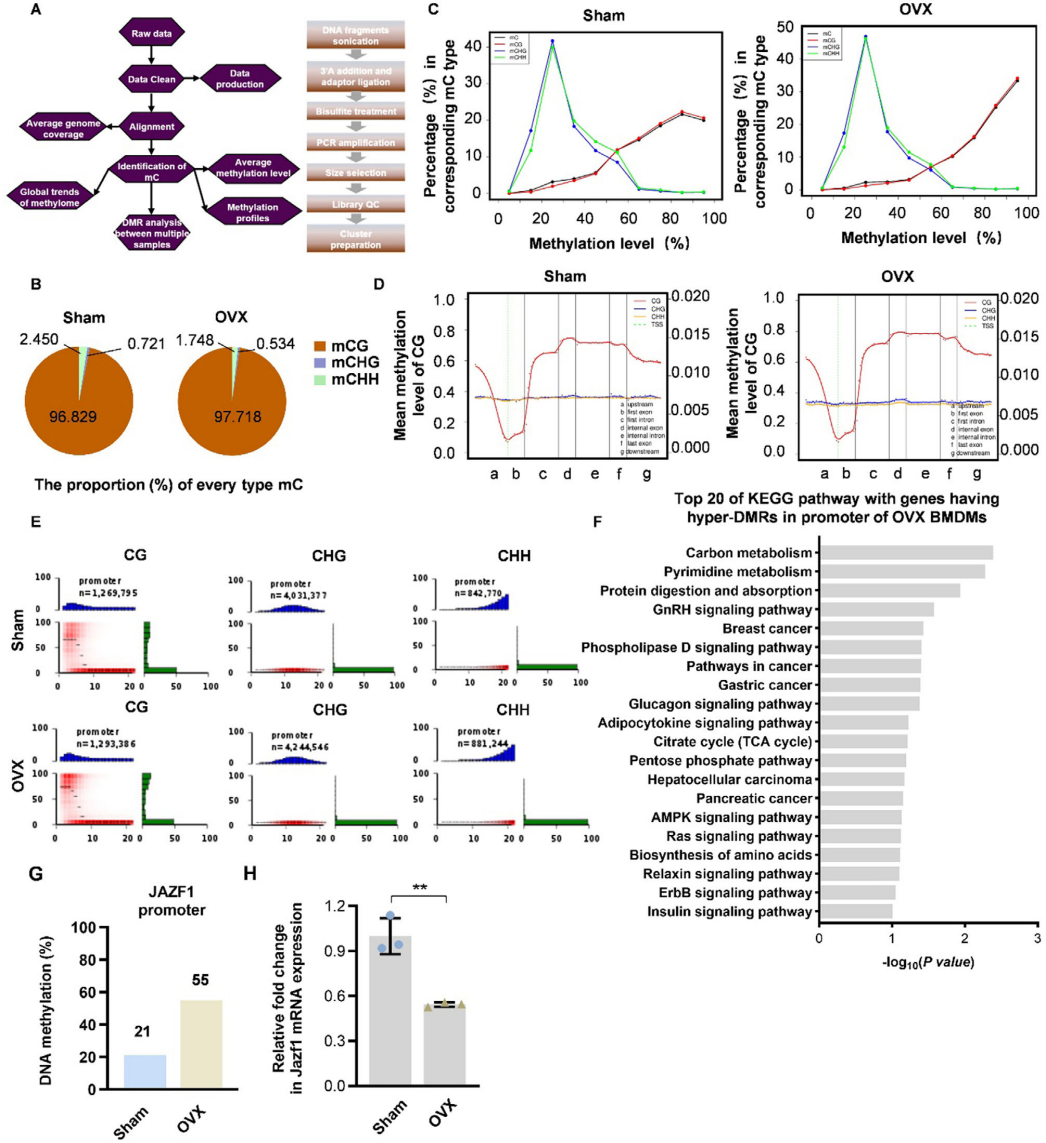
DNA methylation sequencing revealed differential methylation levels in BMDMs between Sham and OVX mice. A) Procedures of DNA methylation level profiling. B) The proportion of every type of mC in BMDMs from Sham and OVX mice. C) The proportion of different types of distribution methylation C in BMDMs from the groups above (Black line: mC, Red line: mCG, Blue line: mCHG, Green line: mCHH). D) The distribution of methylation levels in different functional elements in genome of BMDMs from the groups above (a: upstream, b:first exon, c:first intron, d:internal exon, e:internal intron, f:last exon, g: downstream). E) The methylation distribution of the promoter region of the genome, and the corresponding CpG density. In the heat map at left below, CpG density (X‐axis) is defined as the number of CPG in a 200 bp window, and the vertical axis represents the average methylation level of CPG. The thin black line shows the median methylation level for a given CpG density in such a window. The red areas, from light to dark, indicate the number of CPG at a particular methylation level and CpG density. The blue bar at the top shows the distribution of CpG density, mapped on the horizontal axis, and the green bar at the right shows the distribution of methylation levels, mapped on the vertical axis. F) KEGG pathway analysis of genes having hyper‐DMRs in the promoter of OVX BMDMs. The top 20 KEGG pathways were shown. G) The ratio of DNA methylation in the promoter of the *Jazf1* gene. H) Differential expression of selected *Jazf1* gene in macrophages derived from Sham or OVX mice was validated by qPCR. Data represent mean ± SD. **P <0.01 (Student's t test).

Based on the above findings, we focused on the reasons for dysfunctional macrophages under estrogen deficiency. Considering estrogen deficiency as an environmental factor, it is plausible to hypothesize that DNA methylation serves as a primary epigenetic regulatory mechanism for environmental factors and chronic inflammatory regulation. Since hormonal alterations regulate epigenetics, we detected differential DNA methylation in macrophages from Sham and OVX mice (**Figure** **4**A). The proportion of methylated CpGs and the average methylation level were shown (Figure 4B,C). Comparisons of methylated C levels between different gene regions and functional elements revealed that both groups presented hypomethylation in the promoter region (Figure 4D). Interestingly, total methylated CG, CHG, and CHH frequencies in the promoter region were higher in OVX‐BMDMs than the control (Figure 4E). The methylation levels of every type C in the chromosome or in different functional elements in genome of Sham‐BMDMs and OVX‐BMDMs were shown in Tables S1 and S2 (Supporting Information), respectively. The results of Kyoto Encyclopedia of Genes and Genomes (KEGG) pathway analysis indicated that genes having hyper‐DMRs in their promoters are mainly involved in 20 pathways, including Carbon metabolism, Pyrimidine metabolism, Protein digestion and absorption, GnRH signaling pathway, Breast cancer, Phospholipase D signaling pathway, Pathways in Cancer, Gastric cancer, Glucagon signaling pathway, Adipocytokine signaling pathway, Citrate cycle (TCA cycle), Pentose phosphate pathway, Hepatocellular carcinoma, Pancreatic cancer, AMPK signaling pathway, Ras signaling pathway, Biosynthesis of amino acids, Relaxin signaling pathway, ErbB signaling pathway and Insulin signaling pathway (Figure 4F). Then we further identified the top 30 genes exhibiting the most significant differences in DNA methylation (Table S3, Supporting Information). Notably, the *Jazf1* gene emerged as closely associated with adipose tissue and macrophages. Among the DMR genes, the DNA methylation ratio of *Jazf1* promoter reached 55% in the OVX group compared with 21% in the control group (Figure 4G). Subsequently, we validated the differential expression of the *Jazf1* gene in macrophages from Sham and OVX mice using qPCR. As anticipated, the mRNA expression level of *Jazf1* was reduced in the OVX group (Figure 4H). These findings suggest that estrogen deficiency alters DNA methylation levels in macrophages, and JAZF1 may serve as a potential therapeutic target.

### 2.5 Hypermethylation of the Jazf1 Promoter Enhances Pro‐Inflammatory Changes in Macrophages

**Figure 5:**
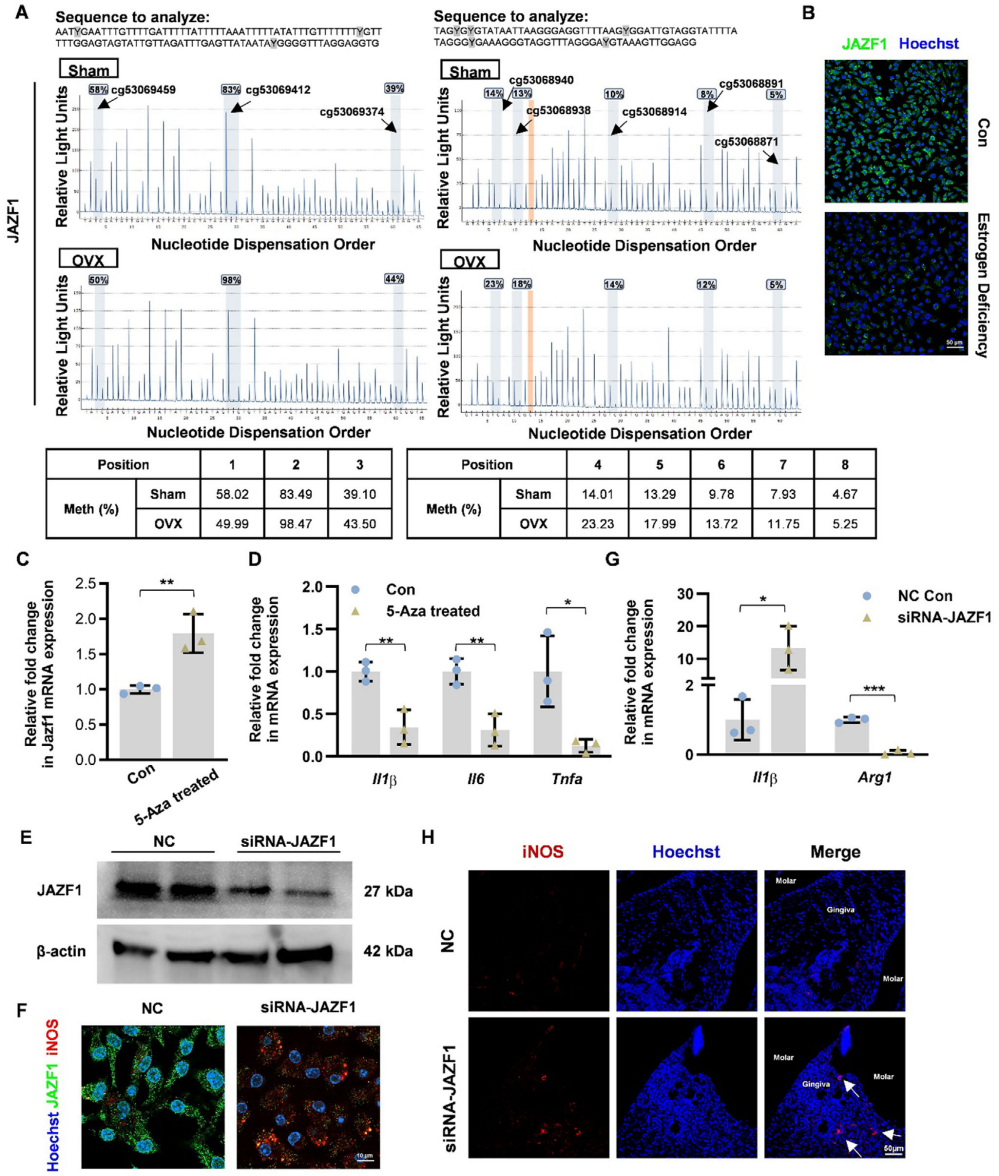
DNA methylation of Jazf1 regulates macrophage polarization. A) Representative images of pyrosequencing for *Jazf1* gene in macrophages derived from Sham or OVX mice. Eight methylation sites were identified using pyrosequencing for target‐specific analysis in the JAZF1 promoter region. B) Representative immunofluorescence of the macrophages cultured in control or estrogen deficient condition, stained for JAZF1 (green) and Hoechst (blue). C) The mRNA expression levels of *Jazf1* in macrophages with (+) or without (‐) the 5‐Aza‐2′‐deoxycytidine treatment. Aza (‐) and Aza (+) were abbreviated by 5‐Aza‐2′‐deoxycytidine (‐) and 5‐Aza‐2′‐deoxycytidine (+), respectively. D) The mRNA expression levels of *Tnfα, Il6,Il1β* genes in macrophages with (+) or without (‐) the 5‐Aza‐2′‐deoxycytidine treatment. E) Levels of JAZF1 protein in macrophages following JAZF1 knockdown were detected by western blot. F) Immunofluorescence detection of iNOS and JAZF1 expression following transfection with JAZF1 siRNA or NC control. G) The mRNA expression levels of *Il1β* and *Arg1* genes in macrophages after JAZF1‐siRNA or NC transfection. Data represent mean ± SD. *P <0.05, **P <0.001 (Student t‐test). H) The expression of iNOS in periodontal tissue of mice injected with JAZF1‐siRNA or NC was detected using immunofluorescence staining.

To corroborate the hypermethylation of the *Jazf1* promoter in macrophages derived from ovariectomized (OVX) mice, as indicated by whole‐genome bisulfite sequencing (WGBS) methylation array results, we conducted an analysis of eight CpG sites within the *Jazf1* promoter using pyrosequencing. This method is considered the gold standard for assessing methylation levels due to its precision and reproducibility. Among these sites, seven CpG positions (cg53069459, cg53069412, cg53069374, cg53068940, cg53068938, cg53068914, cg53068891, cg53068871) exhibited significant hypermethylation in the *Jazf1* promoter of macrophages from the OVX group (**Figure** **5**A). Immunofluorescence analysis revealed that JAZF1 expression was markedly reduced in macrophages cultured under estrogen‐deficient conditions compared to the control group (Figure 5B). To further elucidate the impact of *Jazf1* promoter methylation on its expression, we examined the mRNA expression levels of *Jazf1* in estrogen‐deficient macrophages, both with and without treatment with 5‐Aza‐2′‐deoxycytidine. The results demonstrated a significant increase in *Jazf1* expression following demethylation treatment in estrogen‐deficient macrophages (Figure 5C). These findings suggest that hypermethylation of the Jazf1 promoter may serve as a potential mechanism underlying the aberrantly low expression of Jazf1 in macrophages under conditions of estrogen deficiency.

Juxtaposed with another zinc finger (Znf) protein 1 (JAZF1, also known as TIP27, ZNF802) encodes a 27 kDa protein with three C2H2‐type ZnFs and functions as a repressor of DNA response element 1 (DR1)‐dependent transcription of NR2C2, TR4.^[^ ^29^ ^]^ JAZF1 is integral to glucose homeostasis and the pathophysiology of metabolic diseases. It is ubiquitously expressed across various tissues, with particularly high expression levels in adipose tissues (AT). JAZF1 mitigates inflammation and immune cell infiltration by attenuating the NF‐κB pathway.^[^ ^25^ ^]^ Studies have demonstrated that JAZF1 reduces CD11c+ adipose tissue macrophages and suppresses the secretion of TNF‐α and IL‐1β, thereby diminishing adipose tissue inflammation in diabetic mice.^[^ ^26^ ^]^ To evaluate our hypothesis, we analyzed the mRNA expression levels of *Tnf‐α*, *Il6*, and *Il1β* in estrogen‐deficient macrophages treated with (+) or without (‐) 5‐Aza‐2′‐deoxycytidine. The expression levels were significantly reduced following the inhibition of DNA methylation by 5‐Aza‐2′‐deoxycytidine (5‐Aza) (Figure 5D). Subsequently, we assessed the role of JAZF1 in macrophage inflammation using JAZF1‐siRNA or negative control. Western blot analysis was employed to confirm the knockdown efficiency of JAZF1 (Figure 5E). Immunofluorescence analysis revealed a significant reduction in JAZF1 expression in macrophages treated with JAZF1‐siRNA compared to the control group. Conversely, JAZF1‐knockdown led to a notable increase in iNOS expression (Figure 5F). Subsequent qPCR analysis confirmed that JAZF1‐knockdown resulted in elevated *Il1β* expression and reduced *Arg1* expression (Figure 5G). Furthermore, in a mouse model, iNOS expression in periodontal tissues was observed to increase following periodontal injection of JAZF1‐siRNA, as compared to the negative control injection group (Figure 5H). Collectively, these findings suggest that hypermethylation of the JAZF1 promoter suppresses its expression and it is likely to promote pro‐inflammatory changes in macrophages.

### 2.6 M1 Macrophage‐Derived sEVs Promoted M1 Macrophage Polarization

Previous studies have demonstrated that small extracellular vesicles (sEVs) are critical in cellular communication and regulation of distant organs. Our prior research successfully isolated, extracted, and characterized macrophage‐derived extracellular vesicles.^[^ ^3^ ^]^ The characteriazation of small extracellular vesicles was also conducted in this study via the Transmission electron microscopy (TEM), Nanoflow cytometry (nanoFCM) and Western Blot analysis of CD63 and CD81 marker (Figure S1, Supporting Information). To investigate the potential connection between adipose tissue and periodontitis, small extracellular vesicles were isolated from visceral adipose tissue (AD‐sEVs) and administered intravenously into mice following pretreatment with DiR fluorescent dye (Figure S2A, Supporting Information). The in vivo imaging results implied that AD‐sEVs could reach periodontium via circulation (Figure S2B, Supporting Information). Further vesicle‐tracing experiments revealed that DiO‐labeled M1‐sEVs could be effectively endocytosed by macrophages, as shown by immunofluorescence and flow cytometry (Figure S2C, Supporting Information). Moreover, M1‐sEVs derived from M1‐polarized macrophages cultured in estrogen deficiency or in control conditions presented similar uptake efficiency (Figure S2D,E, Supporting Information).

**Figure 6:**
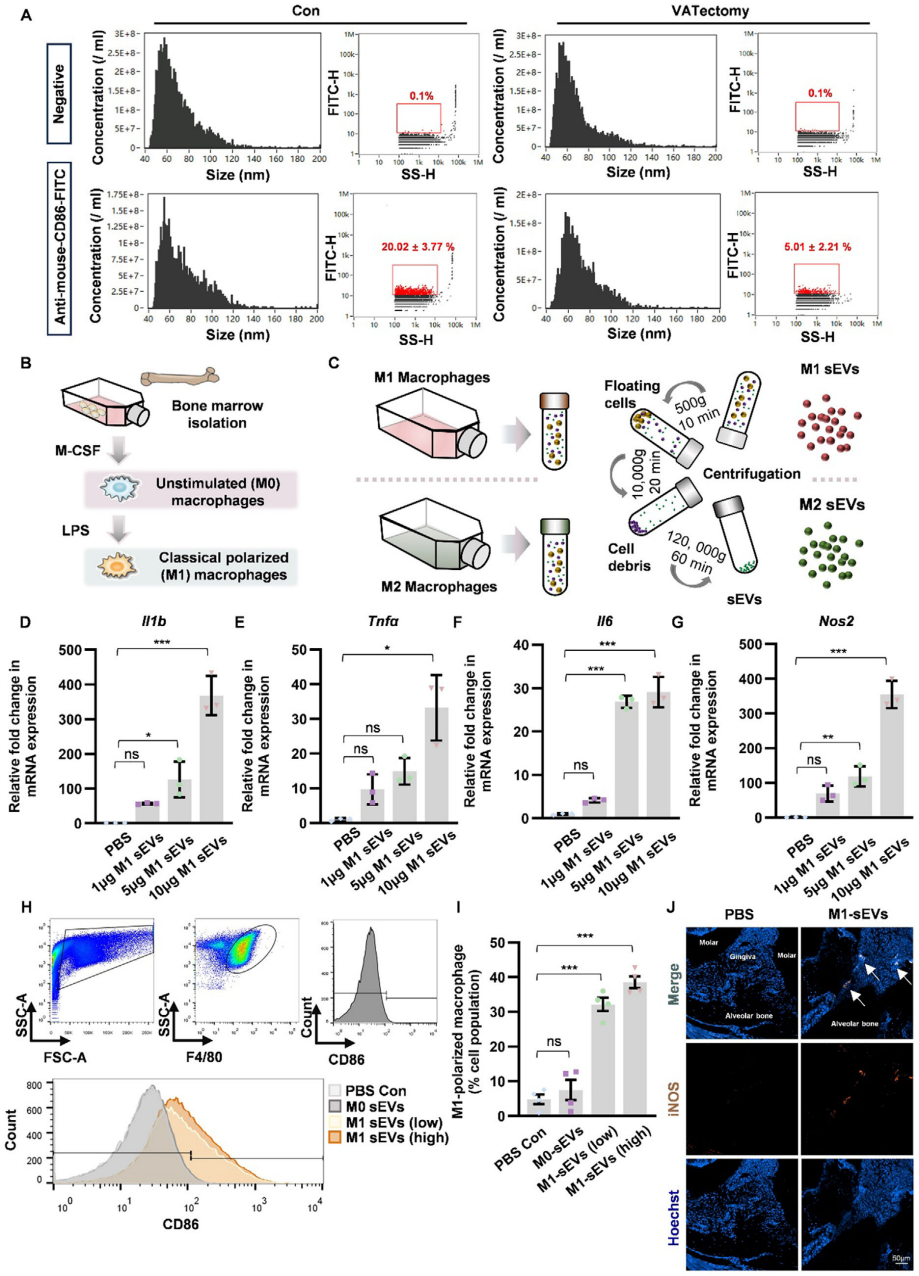
Extracellular vesicles derived from M1 macrophages promoted M1 polarization. A) Small extracellular vesicles isolated from the serum of OVX mice with VATectomy (n = 6) or control treatment (n = 5) were analyzed the ratio of CD86‐positive via nanoflow cytometry (nanoFCM). Data represent mean ± SD. B,C) A schematic illustration shows bone marrow isolation, macrophage induction, stimulation and EV‐isolation. D–G) After 6 h of incubation with 1, 5,10 µg M1‐sEVs or an equal volume of PBS, the mRNA expression levels of the M1 characteristic gene profile, including *Il‐1β*, *Il6*, *Tnf‐α* and *Nos2*, were measured. H,I) Representative gates and corresponding group data for identifying F4/80+ CD86+ cells in BMDMs after treatment of 10 µg (low) or 50 µg (high) M1‐sEVs, 10 µg M0‐sEVs, or PBS control. Data represent mean ± SD. *P < 0.05, **P < 0.01, ***P < 0.001, ns: not significant. (One‐way ANOVA). J) The expression of iNOS in periodontal tissue of mice injected with M1‐sEVs or PBS was detected using immunofluorescence staining.

To explore the relationship between viseral adipose tissue and circulating M1‐sEVs, we further conducted VAT‐resection or control treatment on ovariectomized (OVX) mice, subsequently isolating small extracellular vesicles (sEVs) from the serum. The expression of the M1 macrophage marker CD86 in serum sEVs was quantified via nano‐flow cytometry to assess the levels of M1‐sEVs. The experiments demonstrate a significant reduction in circulating M1‐sEV levels following the excision of visceral adipose tissue in OVX mice (**Figure** **6**A). To further investigate the effect of M1‐sEVs, macrophages in resting state (M0) were co‐cultured with M1‐sEVs (Figure 6B,C). The data of qPCR analysis showed that M1‐sEVs up‐regulated the mRNA expression level of M1 characteristic genes, including *Il1b*, *Tnfα*, *Il6* and *Nos2* (Figure 6D–G). Consistently, FACS detection also revealed that M1‐sEVs induced M1‐like macrophage polarization (Figure 6H,I). In vivo experiments also verified the iNOS‐positive M1‐polarized macropahges increased in periodontal tissues following M1‐sEVs injection (Figure 6J). Taken together, the above results indicated that M1 macrophage‐derived sEVs might potentiate the M1 pro‐inflammatory phenotype after being endocytosed by quiescent macrophages.

### 2.7 miR‐30e‐5p Contained in M1‐sEVs Induced Pro‐Inflammatory Polarization

**Figure 7:**
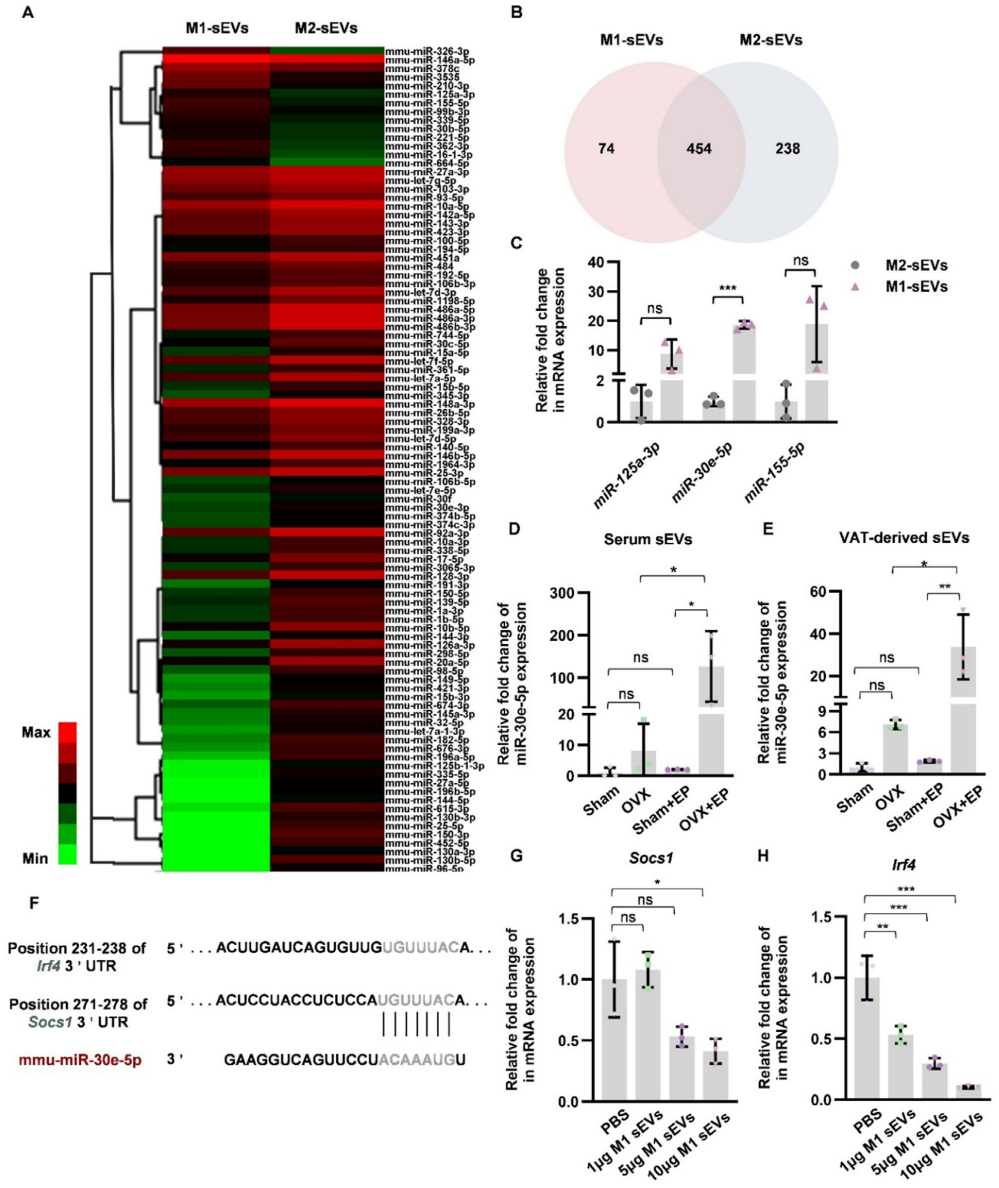
Profiling of miRNAs derived from small extracellular vesicles of M1/M2 macrophages. A) A heatmap of differential miRNA expression between M1‐sEVs and M2‐sEVs, ranging from red (upregulated) to green (downregulated). B) Venn diagram of the miRNAs’ expression in M1‐sEVs and M2‐sEVs. C) qPCR analysis of the level of miRNA expression in M1‐sEVs and M2‐sEVs. Data represent mean ± SD, ns: not significant, ***P <0.001 (Student's t test). D) qPCR analysis of different miR‐30e‐5p expression in serum‐derived‐sEVs from Sham, Sham+EP, OVX and OVX+EP mice. E) qPCR analysis of miR‐30e‐5p expression in sEVs derived from abdominal adipose tissue in the groups above. F) Schema of predicted miRNA‐binding sites at the 3′ UTR region of *Irf4* and *Socs1* genes. G,H) qPCR was used to detect the mRNA levels of the *Socs1* and *Irf4* genes in macrophages after 6 h of incubation with 1, 5, and 10 µg M1‐sEVs or an equal volume of PBS. Data represent mean ± SD, ns: not significant, *P <0.05, **P <0.01, ***P <0.001 (One‐way ANOVA).

Further miRNA sequencing was applied to find out the mechanism of the pro‐inflammatory role of M1‐sEVs. Using miRNA expression levels as a basis for coloring, a heatmap is created to present the different miRNA profiles between M1‐sEVs and M2‐sEVs (**Figure** **7**A). The Venn diagram showed differences and overlaps of detected miRNAs between M1‐sEVs and M2‐sEVs (Figure 7B). We confirmed the validity of miRNA profiling via further qPCR detection (Figure 7C). Among them, miR‐30e‐5p, which is highly expressed in M1‐sEVs, was also found to largely increase in serum derived sEVs (Figure 7D) and AD‐sEVs (Figure 7E) from OVX+EP mice. The TargetScan database provided target gene predictions for miR‐30e‐5p. According to the data, two immune‐related genes, *Irf4* and *Socs1* (Figure 7F), were potential targets of miR‐30e‐5p. Further qPCR analysis showed that the mRNA expression levels of *Socs1* and *Irf4* decreased after treatment of M1‐sEVs (Figure 7G,H).

**Figure 8:**
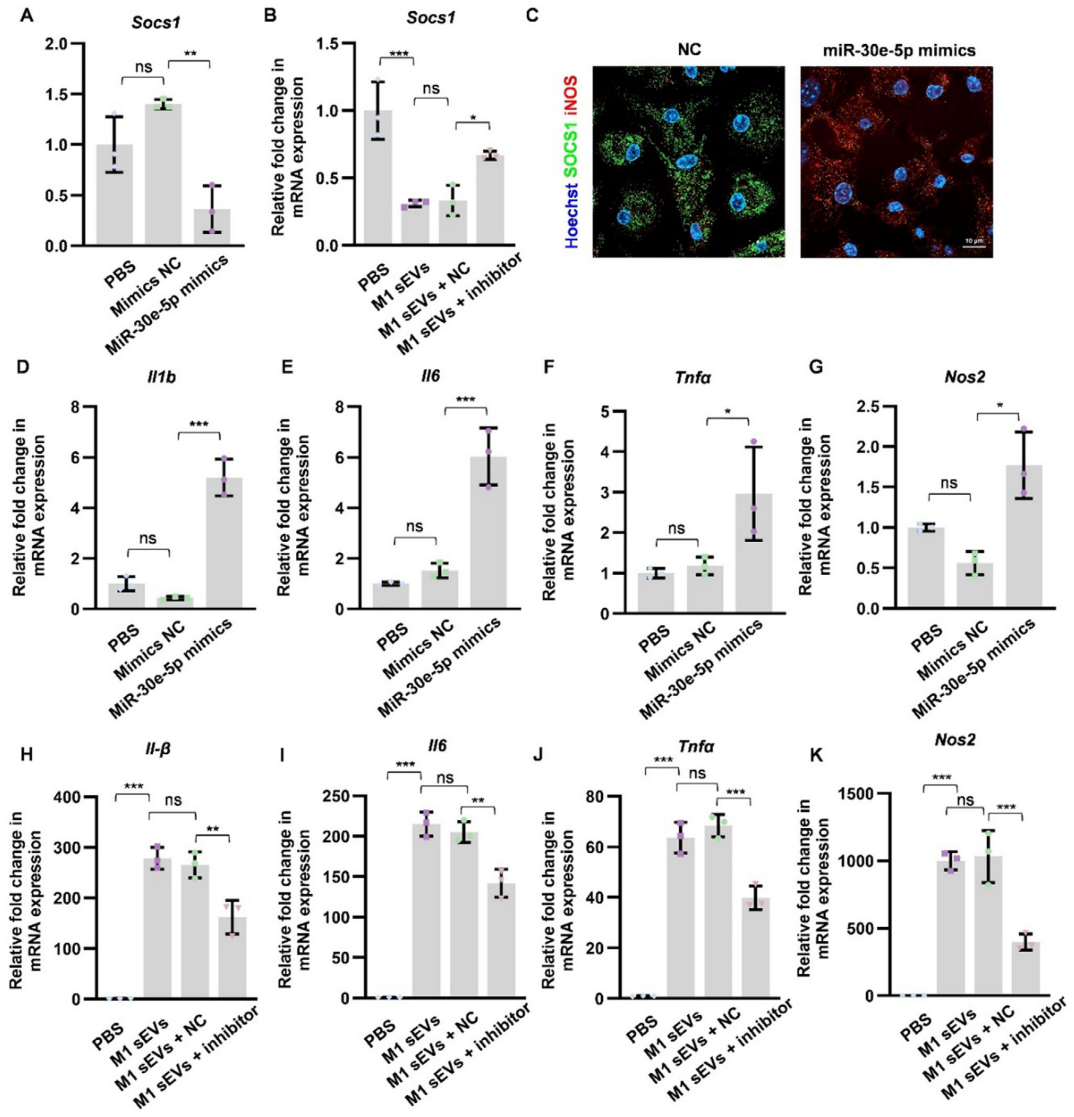
miR‐30e‐5p promoted M1 polarization via targeting *Socs1*. A) The mRNA levels of Socs1 gene in macrophages were detected after 6 h incubation of NC, miR‐30e‐5p mimics or equal volume of PBS by qPCR. B) The mRNA levels of *Socs1* genes in macrophages were detected after 6 h of incubation. C) Immunofluorescence analysis of iNOS and SOCS1 expression following transfection with miR‐30e‐5p mimics or NC. D–G) The mRNA levels of *Il1β*, *Il6*, *Tnfα* and *Nos2* genes in macrophages were detected after 6 h of incubation with NC, miR‐30e‐5p mimics, or an equal volume of PBS by qPCR. H–K) The mRNA levels of *Il1β*, *Il6*, *Tnfα* and *Nos2* genes in macrophages were detected after 6 h incubation of M1‐sEVs, M1‐sEVs + NC, M1‐sEVs + miR‐30e‐5p inhibitor, or an equal volume of PBS by qPCR. Data represents mean ± SD. ns, not significant, *P <0.05, **P <0.01, ***P <0.001 (One‐way ANOVA).

M1‐sEVs were electroporated with either miR‐30e‐5p mimics or inhibitors and subsequently introduced to macrophages to assess the mRNA expression levels of miRNA targets and pro‐inflammatory genes. The expression levels of the miRNA target, *Socs1*, were found to decrease following the overexpression of miR‐30e‐5p (**Figure** **8**A). Conversely, its expression in recipient cells was restored upon treatment with M1‐sEVs electroporated with the miR‐30e‐5p inhibitor (Figure 8B). Immunofluorescence analysis revealed a decrease in SOCS1 expression and an increase in iNOS expression subsequent to miR‐30e‐5p overexpression (Figure 8C). Furthermore, in recipient macrophages, M1‐associated genes, including *Il1b*, *Il6*, *Tnfα*, and *Nos2*, were upregulated following incubation with sEVs pre‐treated with miR‐30e‐5p mimics (Figure 8D–G). These genes were subsequently downregulated when co‐cultured with inhibitor‐loaded M1‐sEVs (Figure 8H–K). Collectively, these findings suggest that miR‐30e‐5p in M1‐sEVs may facilitate M1‐like macrophage polarization by modulating *Socs1* expression. This study highlights the role of adipose tissue macrophages as initiators of exacerbated periodontitis in estrogen‐deficient environments through the action of amplifier extracellular vesicles and its loading miR‐30e‐5p.

## 3 Discussion

During our experiments, we observed that OVX mice exhibited a phenotype characterized by the accumulation of visceral adipose tissue. Furthermore, we identified abdominal obesity, a marker of visceral fat accumulation,^[^ ^24^ ^]^ as a prominent characteristic in postmenopausal women^[^ ^25^, ^26^, ^27^ ^]^ and correlated with periodontitis. Utilizing the OVX mouse model for periodontitis, we discovered that estrogen deficiency led to an increase in M1‐polarized macrophages within the visceral adipose tissue of OVX mice with periodontitis. Additionally, small extracellular vesicles derived from M1‐polarized macrophages facilitated systemic M1‐like pro‐inflammatory polarization and induced excessive M1‐macrophage infiltration, through delivering pro‐inflammatory miRNAs, including miR‐30e‐5p. The upstream mechanism of the dysfunctional macrophage status might result from the abnormal adipose microenvironment caused by hyper DNA methylation of the promoter of the *Jazf1* gene in macrophages under estrogen deficiency.

Previous epidemiological evidence demonstrates that periodontal diseases promote inflammation in adipose tissue, leading to diabetes and obesity.^[^ ^30^ ^]^ However, the majority of prior research has predominantly concentrated on the association between obesity and inflammation. Distinct from these earlier studies, our research introduces a novel perspective by examining sex‐specific obesity, particularly abdominal obesity induced by estrogen deficiency. We explore the epigenetic alterations in adipose tissue macrophages within this unique environmental context. Furthermore, we investigate the mechanisms underlying the transition from systemic pro‐inflammatory states to sub‐health conditions, integrating findings from both animal experiments and clinical sample analyses. This study suggests that periodontal pathogens serve merely as the initial trigger for a cascade of inflammatory responses. The exacerbation of inflammation and the progression of periodontitis are primarily attributed to a “sub‐health” state of chronic inflammation throughout the body, which is exacerbated by estrogen deficiency. This “sub‐health state” is analogous to the heightened susceptibility to acne and inflammation observed in women during puberty,^[^ ^31^ ^]^ when elevated estrogen levels render the skin more reactive to bacterial irritation, representing a “pre‐disease state”.^[^ ^32^, ^33^ ^]^ Consequently, this condition elucidates our findings that not all patients with estrogen deficiency will inevitably experience exacerbation of periodontitis. Although there is a general positive correlation, some individuals maintain a healthy periodontal status. This discrepancy may be attributed to effective oral hygiene practices in certain patients, which prevent the activation of periodontal pathogens as a critical trigger.

Prior research has primarily concentrated on the direct impact of estrogen deficiency on osteoclasts and osteoblasts. However, clinical investigations have revealed phenomena, such as heightened inflammation in the periodontal tissues of menopausal women, that cannot be solely attributed to osteoclast activity.^[^ ^34^, ^35^, ^36^ ^]^ Recent studies have demonstrated that estrogen deficiency can modify the immune‐inflammatory response, resulting in heightened inflammation and an increased susceptibility to infections.^[^ ^37^ ^]^ Our study identified an elevation in macrophage inflammation in estrogen‐deficient mice, alongside an increased pro‐inflammatory phenotype of macrophages in both adipose and periodontal tissues. Consistently, evidence suggests that estrogen promotes M2 macrophage polarization and facilitates browning in adipose tissue.^[^ ^38^ ^]^ In addition, a study found that estrogen deficiency caused M1 macrophage polarization and promoted steatohepatitis in obese OVX mice.^[^ ^39^ ^]^

Given that hormonal changes belong to environmental stimuli, might regulate gene mainly through an epigenetic way, we subsequently investigated the upstream mechanism of how estrogen deficiency initially altered adipose tissue macrophages from the perspective of epigenetics and conducted DNA methylation sequencing. Notably, among the differentially methylated region (DMR) genes, we discovered that the promoter region of *Jazf1* exhibited hypermethylation in estrogen deficiency. JAZF1 (juxtaposed with another zinc finger protein 1), also known as TIP27 (TAK1‐interacting protein 27) or ZNF802 (zinc finger protein 802), is a 243 amino acid protein that localizes to the nucleus and contains three C2H2‐type zinc fingers. It interacts with the nuclear orphan receptor TR4 and is thought to function as a transcriptional repressor. Previous investigations have established that JAZF1 suppresses inflammation (including IL‐1β, IL‐4, IL‐6, IL‐8, IL‐10, TNFα, IFN‐γ, IAR‐20, COL3A1, laminin, and MCP‐1) by reducing NF‐κB pathway activation and AT immune cell infiltration.^[^ ^40^, ^41^ ^]^  Meng et al. found that overexpression of JAZF1 decreased ATM numbers and pro‐inflammatory cytokines secretion, indicating that JAZF1 reduces adipose tissue inflammation via regulating macrophages.^[^ ^42^ ^]^ Furthermore, JAZF1, commonly referred to as the diabetes gene, plays a critical role in regulating metabolism and inhibiting lipid accumulation. Dysregulated expression of JAZF1 has been implicated in the development of type 2 diabetes.^[^ ^29^, ^43^ ^]^ Among the eight CpG sites validated by pyrosequencing, one site in the OVX group showed lower methylation than in the Sham group, which contradicts the general trend of increased methylation in the gene's promoter region identified by WGBS. This discrepancy highlights the regional and site‐specific characteristics of DNA methylation. WGBS provides an average methylation level across all CpG sites in a region, indicating a general increase in methylation in the JAZF1 promoter in the OVX group, while pyrosequencing offers precise measurements at specific sites, revealing that methylation can vary significantly within a region. Additionally, treatment of macrophages in the OVX group with the methylation inhibitor 5‐Aza revealed changes in the expression of JAZF1 and macrophage polarization genes, indirectly supporting the role of JAZF1 methylation in regulating macrophage polarization. Our research has additionally demonstrated that DNA hypermethylation of JAZF1 might contribute to inflammation and promote M1 macrophage polarization.

Our study further demonstrated that the accumulation of visceral adipose tissue and macrophage inflammation under estrogen‐deficient conditions may exacerbate systemic chronic inflammation via small extracellular vesicles (sEVs). Consistent with previous research, extracellular vesicles derived from macrophages have been described to regulate inflammation and affect distant tissues.^[^ ^44^, ^45^ ^]^ Exosomes from IL‐4 polarized macrophages, for example, have been linked to the control of cardiometabolic inflammation and diabetes. Reprogramming circulating monocytes reduced systematic inflammation in the circulation, aorta, adipose tissue, and the liver.^[^ ^46^ ^]^ Furthermore, the macrophage‐derived sEVs would target macrophages more efficiently. It has been reported that exosomes from visceral‐fat‐tissues express lower CD47 and thus facilitate their endocytosis by macrophages.^[^ ^23^ ^]^

To investigate the mechanism, we newly characterized a specific miRNA, miR‐30e‐5p, that could promote pro‐inflammatory M1 polarization. Notably, miR‐30e‐5p was predominantly expressed and abundant in macrophages.^[^ ^3^, ^47^, ^48^ ^]^ In addition, we demonstrated that miR‐30e‐5p suppresses the expression of *Socs1*, a gene recognized for its anti‐inflammatory regulatory functions. It has been reported that downregulated expression of SOCS1 enhances M1‐type polarization, probably via activating the JAK1/STAT1 pathway.^[^ ^49^ ^]^ Furthermore, exosomal miR‐221 promotes M1 macrophage polarization via SOCS1/STATs to promote inflammatory response.^[^ ^50^ ^]^ Meanwhile, IRF4 is considered a key regulator of anti‐inflammation and M2 macrophage polarization.^[^ ^51^, ^52^ ^]^ Richa et al. identified that the upregulated miR‐30e‐5p promote inflammation by targeting key negative regulators such as *SOCS1* and *SOCS3* of innate immune signaling pathways.^[^ ^48^ ^]^

In conclusion, our study highlights that during non‐resting states, such as pathogen invasion, adipose tissue serves as a primary site for macrophage accumulation, second only to the bone marrow. Macrophages derived from mature monocytes, characterized by F4/80 positivity, predominate in this context. This study found that in an estrogen‐deficient environment, macrophages become pro‐inflammatory, increasing M1‐sEVs in circulation. These M1‐sEVs could further transform macrophages, and when periodontal pathogens stimulate and motivate monocytes‐macrophages, it worsens local periodontal macrophage polarization, prolonging inflammation. In summary, we propose that while periodontal pathogens trigger inflammation, the chronic “sub‐health” state from low estrogen post‐menopause amplifies inflammation, explaining why some women develop aggravated periodontitis while others remain healthy.

## 4 Conclusion

This study found that most ovariectomized (OVX) mice developed significant visceral fat, similar to the abdominal fat increase seen in postmenopausal women linked to periodontitis progression. Removing visceral adipose tissues (VATectomy) reduced periodontitis symptoms in OVX mice. The research revealed that dysfunctional macrophages in VAT, caused by hyper DNA methylation of the *Jazf1* gene promoter under estrogen deficiency, could probably lead to a pro‐inflammatory state. JAZF1‐knockdown resulted in M1‐polarization of macrophages. Additionally, small extracellular vesicles from these M1‐polarized macrophages promoted systemic inflammation by delivering pro‐inflammatory miRNAs like miR‐30e‐5p. These M1‐sEVs could further transform macrophages, and when periodontal pathogens stimulate and motivate monocytes‐macrophages, it worsens local periodontal macrophage polarization, prolonging inflammation. Generally, our study proposes a novel perspective: periodontal pathogens act as a trigger for inflammatory responses, but the real issue is chronic systemic inflammation, worsened by estrogen deficiency, which exacerbates periodontitis. M1‐sEVs can transform macrophages, and when periodontal pathogens activate these cells, it leads to prolonged local inflammation. Pro‐inflammatory macrophages in adipose tissue release sEVs, which activate quiescent macrophages at infection sites, causing persistent inflammation. Addressing the root causes of this dysregulation may offer novel therapeutic strategies for the treatment of periodontitis and other systemic inflammatory conditions, particularly in postmenopausal women.

## 5 Experimental Section

### Ethical Consent

The study  was approved by the Medical Ethics Committee of Hospital of Stomatology Sun Yat‐sen University (No. KQEC‐2023‐13‐0) and the Clinical Research Center of Hospital of Stomatology Sun Yat‐sen University (No. GHKQ‐2002301‐L2).

### Participants and Criteria Design

Women who visited the Hospital of Stomatology at Sun Yat‐sen University and consented to complete the study were eligible for participation. A total of 58 women were recruited, with data collected on the timing of menopause and periodontal assessment. Eligibility required the presence of amenorrhea for 12 consecutive months without any evident pathological changes or physiological causes. The cohort consisted of 26 premenopausal women (aged >36 years) and 33 postmenopausal women (aged ≤65 years). The following conditions were grounds for exclusion: 1) Pregnancy or a history of abdominal tumors, liver disease, kidney disease, colon disease, retroperitoneal disease, or other conditions causing abdominal enlargement. 2) A history of periodontitis treatment within the past year. 3) Diabetes, smoking, leukemia, dental trauma, and the use of nitrobenzene and phenytoin, which may exacerbate periodontitis. 4) Diseases affecting bone metabolism, rheumatism, and immune‐related disorders. The number of subjects enrolled in each group, as well as their average age, height, and weight were listed in Appendix Table S6 (Supporting Information).

### Mice

Eight‐week‐old wild‐type female C57BL/6 mice were used and acquired from the laboratory animal center. Experimental periodontitis (EP) and ovariectomy (OVX) models were induced as described in the previous study.^[^ ^3^ ^]^ These animal experiments were certified by the Institutional Animal Care and Use Committee.

### micro‐CT Analysis

For micro‐CT analysis, 4% paraformaldehyde (PFA) was used to fixed the sampled maxillae and Scanco µCT50 scanner (Scanco Medical AG, Brutishly, Switzerland) was further applied for scanning as reported before.^[^ ^53^ ^]^ To assess alveolar bone loss, the distances between the cemento‐enamel junction and the alveolar bone crest were measured at four sites around each maxillary molar using 3D images. The average of 12 site measurements per sample was reported in millimeters. Evaluations included both buccal and palatal sides to comprehensively assess ligature‐induced bone loss.

### Differentiation and Cell Culture of Bone Marrow Derived Macrophages (BMDMs)

Macrophages were differentiated using 20 ng mL^−1^ macrophage colony‐stimulating factor (M‐CSF) after being isolated from bone marrow and cultured with DMEM medium (including 10% FBS and 1% Penicillin and Streptomycin solution). For M1‐polarization, 100 ng mL^−1^ LPS (Sigma, USA) was added, and cells were co‐cultured for 24 h. For estrogen‐deficient culturing, phenol red‐free DMEM medium was used with activated carbon‐adsorbed FBS, adding 17‐β estradiol (10^−9^ m) as a control.

### Macrophage Depletion

Clodronate liposomes or control liposomes (Clophosome, Cat#F70101C‐N, FormuMax) in equal volume were intraperitoneally injected (0.2 mL per time) to clear macrophages. Flow cytometry and immunofluorescence were conducted to detect the depletion efficiency 24 h after injection.

### Adipose Tissue Isolation

Visceral adipose tissue was isolated and cut into small pieces using ophthalmic scissors and placed in DMEM culture medium with 10% BSA and 1% Penicillin–streptomycin. Hemolysin was added to remove the erythrocytes.

### Flow Cytometry Analysis

Visceral adipose tissues and maxillary periodontal tissues were harvested from mice and digested by 2% collagenase I and 1% DNase for 30 min. The above digests were filtered through nylon mesh filters and centrifuged at 800 rpm for 5 min. Precipitation was collected and resuspended in 5% fetal bovine serum (FBS) containing PBS. Then the cells were dyed with Alexa Fluor 488 anti‐mouse F4/80 (Cat# 123120, BioLegend, US), APC anti‐mouse CD11b (Cat# 101212, BioLegend, US) and PE anti‐mouse CD86 (Cat# 105007, BioLegend, US) on the ice for 20 min. After washing with PBS and centrifugation, 300 µL of 7‐AAD Viability Staining Solution (Cat# 420404, BioLegend, US) was added to per tube to detect cell activity. Following washing, centrifugation, and resuspension, a flow cytometer (BD Biosciences) was applied to detect the fluorescent signal of the above samples.

### Immunofluorescence

The visceral adipose tissues were fixed using a fatty tissue fixative solution and embedded in OCT at −80 °C. Meanwhile, the periodontal tissues were fixed with 4% PFA and decalcified with 0.5 m EDTA for 3 days. They were then sliced at a thickness of 10 µm at temperatures below −30 °C. Before staining, the antigen was repaired with an antigen retrieval solution and blocked for 1 h at room temperature in 1% BSA. Then sections were incubated overnight with primary antibodies, including rat‐anti‐mouse anti‐F4/80 antibody (ab16911, Abcam, USA), anti‐CD3 antibody (ab5690, Abcam, USA), anti‐Myeloperoxidase (MPO) antibody (ab9535, Abcam, USA) and rabbit‐anti‐mouse anti‐MHC antibody (ab180779, Abcam, USA). On the next day, they were washed with PBS three times and stained with goat‐anti‐rabbit Alexa Fluor 488 or goat‐anti‐rat Alexa Fluor 488 second antibodies. Then the slices were counterstained with Hoechst for 10 min at 25 °C. And laser‐scanning confocal microscopy was used to observe the distribution of fluorescence.

### Isolation of Small Extracellular Vesicles

Visceral adipose tissues were collected and cultured in FBS‐free DMEM medium for 48 h. The supernatant was harvested for further isolation. Furthermore, macrophages were cultured in FBS‐free DMEM medium for 2 days. The supernatants were centrifuged at 500 g for 10 min, 10 000 g for 20 min, and 120 000 g for 60 min. The precipitate was resuspended in PBS and centrifugated at 120 000 g for 30 min. The pellets were stored at −80 °C. The isolation and characterization of sEVs was described in our previous study.^[^ ^3^ ^]^

### Fluorescent Tracing of sEVs

BMDMs were co‐cultured with 5 µg DiO‐labeled sEVs derived from M1 macrophages cultured in estrogen deficiency (M1‐E sEVs) or in control conditions (M1+E sEVs) for 6 h. After washing with sterile PBS, 4% PFA was added for fixation. Then Hoechst dye (1:1000, Hoechst 33342, Beyotime Biotechnology, China) was applied to stain the cell nuclei for 10 min at room temperature. Following thorough washing, the cells were observed under a laser confocal microscope. For in vivo tracing, mice were intravenously injected with 10 µg of DiR‐labeled AD‐sEVs. The distribution of the AD‐sEVs in periodontal tissue was observed via the IVIS Lumina II in vivo imaging system (Caliper Life Sciences, US).

### qPCR Analysis

Total RNA was extracted and reverse‐transcribed into cDNA as reported.^[^ ^3^ ^]^ Then SYBR Green (FastStart Essential DNA Green Master, 06924204001, Roche, USA) was used for qPCR analysis. The Roche Real‐Time PCR System was used to detect mRNA expression levels, which were normalized to *β‐actin* or *Gapdh* mRNA levels. To measure miRNA expression, the miRcute Plus miRNA First‐Strand cDNA kit (TIANGEN, Beijing, China) was applied to reverse‐transcribe RNA samples. The reverse primer (Cat# CD109, TIANGEN, Beijing, China) was used for further qPCR analysis. The primers used are listed in Table S4 (Supporting Information).

### miRNA Profiling

Total RNA samples were qualified and further generated for sequencing libraries. Using HiSeq2500 (Illumina; San Diego, CA) for parallel sequencing, the expression level of miRNAs were detected as described.^[^ ^3^ ^]^ A heatmap of miRNA sequencing data was generated via the software Cluster 3.0 and TreeView. TargetScan (http://www.targetscan.org/) was used for miRNA target prediction.

### Genome‐Wide DNA Methylation Sequencing

For the construction of a whole‐genome bisulfite sequencing library, BMDMs from OVX or Sham mice were lysed in lysis buffer and digested by proteinase K. Three samples for replicates were used for DNA methylation sequencing in each group. Genomic DNA was isolated with the instruction of the DNA extraction kit (TIANGEN, Beijing, China). The purity and concentration of DNA were confirmed by NanoDrop. For library construction, DNA was sonicated to an average length of 300 bp. Then, the fragmented DNA was end‐repaired, tailed with a single “A” base, and ligated with adaptors. Bisulfite treatment was conducted using the ZYMOEZ DNA Methylation‐Gold kit. DNA fragments were recovered and amplified to set up the sequencing library, followed by library size selection. The qualified libraries were further sequenced and analyzed by the Chengdu Life Baseline Technology Company. The Kyoto Encyclopedia of Genes and Genomes (KEGG) pathway enrichment analysis (https://david.ncifcrf.gov/summary.jsp) was used to analyze the DMR gene related signaling pathways.

### Pyrosequencing

To validate promoter hypermethylation in macrophages derived from ovariectomized (OVX) mice, pyrosequencing was employed to quantify methylation levels. Initially, DNA was extracted as previously described, followed by bisulfite conversion. The bisulfite‐converted DNA was purified using the Zymo‐Spin IC Column and subsequently amplified via PCR with specific primers targeting the CpG sites. For PCR amplification, 10–20 ng of bisulfite‐converted DNA was utilized with the TaKaRa EpiTap HS kit. The resulting PCR products were analyzed using the PyroMark Q96 pyrosequencing and quantification platform, following the manufacturer's protocol (Shanghai Biotechnology Corporation). A pyrosequencing assay was specifically designed to target the JAZF1 promoter region at positions chr6:53068124‐53069624, with two pairs of primers developed to assess eight CpG sites. Methylation levels were calculated as the percentage of the methylated (C) allele relative to the unmethylated (T) allele background, using the formula: Methylation level = (mC / (mC + umC)) × 100%. The primers for JAZF1 were as follows:

1) 3 CpGs in the promoter region (sequence AATCGAATTTGCTTTGACTTTCATTTCCAAACCTCTACACTTGCCCCTTTCGTCTTTGGAGCAGCACTGCCAGACCTGAGCCACAACACGGGGTTCAGGAGGTG):

PCR Forward 5′‐ AGAGGTTATTTGGTTAATAGTATGGT‐3′

PCR Reverse 5′‐ TCAATTTCTTTACCCCAAATTCCC‐3′

Sequencing 5′‐GGTGAAATTTTTTTTTTAAGTGAA‐3.

2) 5 CpGs in the promoter region (sequence TAGCGCGCATAACCAAGGGAGGCTCCAAGCGGACTGCAGGCACCTCACAGGGCGAAAGGGCAGGCCCAGGGACGTAAAGTTGGAGG):

PCR Forward 5′‐ GTGTTTTTTTGGGGTGAAAAGTA‐3′

PCR Reverse 5′‐ACAACTCCCCCCTCCAACTT‐3′

Sequencing 5′‐ATAGATGTGTATTTATTAAGAATG‐3′.

### 5‐Aza‐2′‐Deoxycytidine Treatment

Bone marrow derived macrophages (BMDMs) were cultured in phenol red free medium with 10% FBS (after activated carbon adsorption treatment) and 1% Penicillin–streptomycin solution to simulate an estrogen‐deficient environment. Then, cells were treated with DNMT inhibitors 5‐Aza‐2′‐deoxycytidine (5‐Aza, 5 µm) or DMSO as control for 48 h.

### SiRNA Transfection

JAZF1‐siRNA and Negative control (NC) were designed and produced by GenePharma Company. The sequences are listed in Table S5 (Supporting Information). siRNA transfection was performed using siRNA‐mate plus Transfection Reagent. Macrophages were cultured overnight on 6 cm plates. JAZF1 siRNA or NC was incubated with 51 µL of buffer and 5 µL of siRNA (1 OD siRNA diluted with 125 µL DEPC‐treated H2O), and then thoroughly mixed with 9 µL of siRNA transfection reagent for 5 min at room temperature. Subsequently, the mixture was applied to cells on each plate. After 48 h, cells were harvested, and western blot analysis was conducted to verify the knockdown efficiency of JAZF1 using an anti‐JAZF1 antibody (Cat#sc‐376503, Santa Cruz Biotechnology).

### Statistical Analysis

Statistical analyses were conducted via GraphPad Prism (La Jolla, CA, USA). The data were tested for normal distribution. Parametric tests were conducted on data that had a normal distribution, whereas non‐parametric tests were used on data that did not have a normal distribution. Comparisons between two groups were calculated by the Student's t test. Differences at *p* <0.05 were considered statistically significant.

## Conflict of Interest

The authors declare no conflict of interest.

## Author Contributions

J.T. performed conceptualization; D.L., T.Y., and Y.L. methodology; D.L., X.L., C.H., and J.Y. performed investigation; J.T. performed supervision; D.L. wrote the original manuscript; J.T. reviewed and edited the final manuscript.

## Supporting information



Supporting Information

## Data Availability

The data that support the findings of this study are available from the corresponding author upon reasonable request.

## References

[1] D. Trindade , R. Carvalho , V. Machado , L. Chambrone , J. J. Mendes , J. Botelho , J. Clinical Periodontol. 2023, 50, 604. 36631982 10.1111/jcpe.13769

[2] B. Yu , C. Y. Wang , Periodontol 2000 2022, 89, 99. 35244945 10.1111/prd.12422 PMC9067601

[3] D. Li , Y. Liu , X. Lyu , C. Hu , T. Yan , J. Yan , Y. Liao , X. Chen , J. Tan , Chem. Eng. J. 2022, 435, 134870.

[4] Z.‐F. Xiao , J.‐B. He , G.‐Y. Su , M.‐H. Chen , Y. Hou , S.‐D. Chen , D.‐K. Lin , Arthritis Res. Ther. 2018, 20, 207. 30201052 10.1186/s13075-018-1701-1 PMC6131954

[5] F. Pamuk , A. Kantarci , Periodontol 2000 2022, 90, 186. 35916870 10.1111/prd.12457

[6] R. Al Habashneh , W. Azar , A. Shaweesh , Y. Khader , Obesity Res. Clin. Pract. 2016, 10, 15. 10.1016/j.orcp.2015.03.010 25890852

[7] L. Scheja , J. Heeren , Nat. Rev. Endocrinol. 2019, 15, 507. 31296970 10.1038/s41574-019-0230-6

[8] X. Zhou , Z. Li , M. Qi , P. Zhao , Y. Duan , G. Yang , L. Yuan , Theranostics 2020, 10, 8197. 32724466 10.7150/thno.43968 PMC7381731

[9] K. Pafili , S. Kahl , L. Mastrototaro , K. Strassburger , D. Pesta , C. Herder , J. Pützer , B. Dewidar , M. Hendlinger , C. Granata , N. Saatmann , A. Yavas , S. Gancheva , G. Heilmann , I. Esposito , M. Schlensak , M. Roden , J. Hepatol. 2022, 77, 1504. 35988689 10.1016/j.jhep.2022.08.010

[10] D. Mathis , Cell Metab. 2013, 17, 851. 23747244 10.1016/j.cmet.2013.05.008 PMC4264591

[11] M. Boesch , A. Lindhorst , R. Feio‐Azevedo , P. Brescia , A. Silvestri , M. Lannoo , E. Deleus , J. Jaekers , H. Topal , B. Topal , T. Ostyn , M. Wallays , L. Smets , L. Van Melkebeke , A. Härtlova , T. Roskams , P. Bedossa , J. Verbeek , O. Govaere , S. Francque , A. Sifrim , T. Voet , M. Rescigno , M. Gericke , H. Korf , S. van der Merwe , J. Hepatol. 2024, 80, 397. 37977244 10.1016/j.jhep.2023.10.039

[12] A. Michaud , R. Drolet , S. Noël , G. Paris , A. Tchernof , Metabolism 2012, 61, 689. 22154325 10.1016/j.metabol.2011.10.004

[13] V. D. Dixit , Diabetes 2013, 62, 2656. 23881195 10.2337/db13-0608 PMC3717859

[14] H. Kane , L. Lynch , Trends Immunol. 2019, 40, 857. 31399336 10.1016/j.it.2019.07.006

[15] K. E. Chen , N. M. Lainez , M. G. Nair , J. Neuroinfl. 2021, 18, 140. 10.1186/s12974-021-02183-2 PMC8218389 34154608

[16] G. Matacchione , J. Perugini , E. Di Mercurio , J. Sabbatinelli , F. Prattichizzo , M. Senzacqua , G. Storci , C. Dani , G. Lezoche , M. Guerrieri , A. Giordano , M. Bonafè , F. Olivieri , Geroscience 2022, 44, 1941. 35247131 10.1007/s11357-022-00536-0 PMC9616990

[17] L. Boutens , G. J. Hooiveld , S. Dhingra , R. A. Cramer , M. G. Netea , R. Stienstra , Diabetologia 2018, 61, 942. 29333574 10.1007/s00125-017-4526-6 PMC6448980

[18] B. F. Zamarron , T. A. Mergian , K. W. Cho , G. Martinez‐Santibanez , D. Luan , K. Singer , J. L. DelProposto , L. M. Geletka , L. A. Muir , C. N. Lumeng , Diabetes 2017, 66, 392. 28108608 10.2337/db16-0500 PMC5248991

[19] S. Chakarov , C. Blériot , F. Ginhoux , J. Exp. Med. 2022, 219, 20211948. 10.1084/jem.20211948 PMC9098652 35543703

[20] A. R. Johnson , J. J. Milner , L. Makowski , Immunol. Rev. 2012, 249, 218. 22889225 10.1111/j.1600-065X.2012.01151.x PMC3422768

[21] W. Liang , Y. Qi , H. Yi , C. Mao , Q. Meng , H. Wang , C. Zheng , Front Immunol. 2022, 13, 908749. 35757707 10.3389/fimmu.2022.908749 PMC9222901

[22] W. Xia , Y. Liu , X. Jiang , M. Li , S. zheng , Z. Zhang , X. Huang , S. Luo , Y. Khoong , M. Hou , T. Zan , J. Nanobiotechnol. 2023, 21, 128. 10.1186/s12951-023-01869-4 PMC10091677 37046252

[23] M. Wei , X. Gao , L. Liu , Z. Li , Z. Wan , Y. Dong , X. Chen , Y. Niu , J. Zhang , G. Yang , ACS Nano 2020, 14, 5099. 32275391 10.1021/acsnano.0c01860

[24] K. Ono , C. Sogawa , H. Kawai , M. T. Tran , E. A. Taha , Y. Lu , M. W. Oo , Y. Okusha , H. Okamura , S. Ibaragi , M. Takigawa , K.‐I. Kozaki , H. Nagatsuka , A. Sasaki , K. Okamoto , S. K. Calderwood , T. Eguchi , J. Extra. Ves. 2020, 9, 1769373. 10.1080/20013078.2020.1769373 PMC7580842 33144925

[25] W. Sun , C. Xing , L. Zhao , P. Zhao , G. Yang , L. Yuan , Mol. Therapy Nucl. Acids 2020, 20, 558. 10.1016/j.omtn.2020.03.016 PMC7182664 32334416

[26] Q. Ou , L. Tan , Y. Shao , F. Lei , W. Huang , N. Yang , Y. Qu , Z. Cao , L. Niu , Y. Liu , X. Kou , S. Shi , Small 2022, 18, 2200306. 10.1002/smll.202200306 35481721

[27] C. Wang , C. Xing , Z. Li , Y. Liu , Q. Li , Y. Wang , J. Hu , L. Yuan , G. Yang , Bioactive Mater. 2022, 8, 494. 10.1016/j.bioactmat.2021.06.005 PMC8427223 34541415

[28] Z. Zhao , T. Shuang , Y. Gao , F. Lu , J. Zhang , W. He , L. Qu , B. Chen , Q. Hao , Cancer Lett. 2022, 530, 45. 35051533 10.1016/j.canlet.2022.01.011

[29] A. Kobiita , S. Godbersen , E. Araldi , U. Ghoshdastider , M. W. Schmid , G. Spinas , H. Moch , M. Stoffel , Cell Rep. 2020, 32, 107846. 32640216 10.1016/j.celrep.2020.107846

[30] K. Masood Mirza , A. A. Khan , M. M. Ali , S. Chaudhry , Pakistan Diabetes Care 2007, 30, 3046. 17712028 10.2337/dc07-0502

[31] R. H. Straub , Endocrine Rev. 2007, 28, 521. 17640948 10.1210/er.2007-0001

[32] G. Hall , T. J. Phillips , J. American Acad. Dermatol. 2005, 53, 555. 10.1016/j.jaad.2004.08.039 16198774

[33] G. S. Chen , H. L. Lin , C. Y. Wu , Int. J. Dermatol. 2009, 48, 993. 19702988 10.1111/j.1365-4632.2008.04021.x

[34] M. C. Walsh , N. Kim , Y. Kadono , J. Rho , S. Y. Lee , J. Lorenzo , Y. Choi , Annu. Rev. Immunol. 2006, 24, 33. 16551243 10.1146/annurev.immunol.24.021605.090646

[35] G. Mori , P. D'Amelio , R. Faccio , G. Brunetti , Clin. Develop. Immunol. 2013, 2013, 720504. 10.1155/2013/720504 PMC3725924 23935650

[36] S. Cenci , M. N. Weitzmann , C. Roggia , N. Namba , D. Novack , J. Woodring , R. Pacifici , J. Clin. Invest. 2000, 106, 1229. 11086024 10.1172/JCI11066 PMC381439

[37] H. Carlsten , Immunol. Rev. 2005, 208, 194. 16313350 10.1111/j.0105-2896.2005.00326.x

[38] O. J. Sul , H. J. Hyun , M. Rajasekaran , J. H. Suh , H. S. Choi , J. Cell. Physiol. 2021, 236, 1875. 32716106 10.1002/jcp.29971

[39] Z. Shu , G. Zhang , X. Zhu , W. Xiong , Biochem. Biophys. Res. Commun. 2022, 596, 63. 35114586 10.1016/j.bbrc.2022.01.085

[40] W. Chen , Y. Zhong , Y. Yuan , M. Zhu , W. Hu , N. Liu , D. Xing , Genes & Dis. 2023, 10, 2457. 10.1016/j.gendis.2022.10.029 PMC10404878 37554201

[41] F. Meng , P. Hao , H. Du , Z. Zhou , Med. Sci. Monit. Basic Res. 2020, 26, 924124. 10.12659/MSMBR.924124 PMC7377004 32655126

[42] F. Meng , Y. Lin , M. Yang , M. Li , G. Yang , P. Hao , L. Li , Biomed Res. Int. 2018, 2018, 1. 10.1155/2018/4507659 PMC5885486 29765984

[43] L. Li , Y. Yang , G. Yang , C. Lu , M. Yang , H. Liu , H. Zong , Metabolism – Clin. Exper. 2011, 60, 523. 10.1016/j.metabol.2010.04.021 20580384

[44] Y. Yao , Y. Yin , F. Shuai , W. Lam , T. Zhou , Y. Xie , X. He , X. Han , Adv. Sci. 2025, 10.1002/advs.202416159. PMC12302528 40277454

[45] T. Bu , Z. Li , Y. Hou , W. Sun , R. Zhang , L. Zhao , M. Wei , G. Yang , L. Yuan , Theranostics 2021, 11, 9988. 34815799 10.7150/thno.64229 PMC8581418

[46] T. A. Phu , M. Ng , N. K. Vu , L. Bouchareychas , R. L. Raffai , Mol. Therapy : The J. Am. Soc. Gene Therapy 2022, 30, 2274. 10.1016/j.ymthe.2022.03.008 PMC9171286 35292359

[47] K. Miranda , P. Mehrpouya‐Bahrami , P. S. Nagarkatti , M. Nagarkatti , Front Immunol. 2019, 10, 1049. 31134094 10.3389/fimmu.2019.01049 PMC6523050

[48] R. Mishra , P. Krishnamoorthy , H. Kumar , Frontiers in Cellular and Infection Microbiol. 2020, 10, 604016. 10.3389/fcimb.2020.604016 PMC7873556 33585275

[49] Y.‐B. Liang , H. Tang , Z.‐B. Chen , L.‐J. Zeng , J.‐G. Wu , W. Yang , Z.‐Y. Li , Z.‐F. Ma , Mol. Med. Rep. 2017, 16, 6405. 28901399 10.3892/mmr.2017.7384

[50] M. Cai , Y. Shi , T. Zheng , S. Hu , K. Du , A. Ren , X. Jia , S. Chen , J. Wang , S. Lai , Int. Immunopharmacol. 2020, 83, 106493. 32289739 10.1016/j.intimp.2020.106493

[51] L. Hu , S. Li , H. Li , B. Lai , H. Wen , Eur. Surgical Res. 2022, 63, 257. 10.1159/000525753 35780774

[52] J. Yuan , F. Lin , L. Chen , W. Chen , X. Pan , Y. Bai , Y. Cai , H. Lu , Inflammopharmacology 2022, 30, 487. 35235107 10.1007/s10787-022-00942-y

[53] D. Li , Y. Feng , H. Tang , L. Huang , Z. Tong , C. Hu , X. Chen , J. Tan , Front Bioeng. Biotechnol. 2020, 8, 444. 32523937 10.3389/fbioe.2020.00444 PMC7261919

