# Supplementary material for: Adipose Tissue Macrophages as Initiators of Exacerbated Periodontitis in Estrogen‐Deficient Environments via the Amplifier Extracellular Vesicles
Source: Adv Sci (Weinh). 2025 Jul 12;12(37):e06121. doi: 10.1002/advs.202506121 (PMC12499395; doi:10.1002/advs.202506121)
Supplement: Supplementary file 1 — Supporting Information [file ADVS-12-e06121-s001.docx]

Supporting Information

Title •

Adipose Tissue Macrophages as Initiators of Exacerbated Periodontitis in Estrogen-Deficient Environments Via the Amplifier Extracellular Vesicles

Danfeng Li^1,2,3^, Jiayin Yan^1,2,3^, Luxian Chen^1,2,3^, Huiling Li^1,2,3^, Siyuan Ma^1,2,3^, Cheng Hu^1,2,3^, Yuwei Liao^1,2,3^, Jiali Tan^1,2,3^*

1 Hospital of Stomatology, Sun Yat-sen University, Guangzhou 510055, China.

2 Guangdong Provincial Key Laboratory of Stomatology, Guangzhou 510080, China.

3 Guanghua School of Stomatology, Sun Yat-sen University, Guangzhou 510080, China.

E-mail: tanjiali@mail.sysu.edu.cn

**
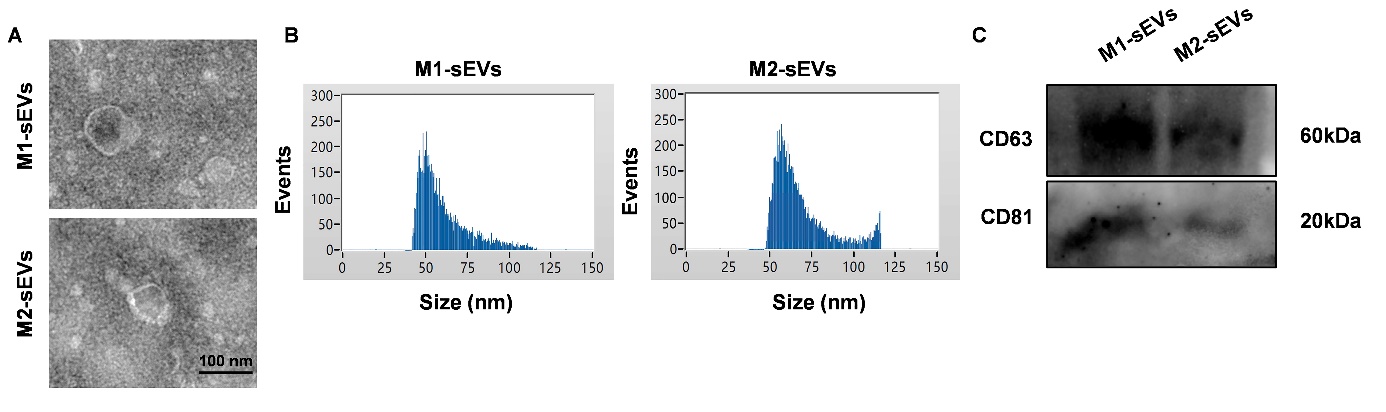
**

**Appendix Figure S1 -** **Characterization of macrophage-derived small extracellular vesicles.**

A Transmission electron microscopy (TEM) of M1-sEVs and M2-sEVs. Scale bar, 100 nm.

B Size distribution of M1-sEVs and M2-sEVs detected by Nanoflow cytometry (nanoFCM).

C Western Blot was used to observe the enrichment of CD63 and CD81 in M1-sEVs and M2-sEVs.

**
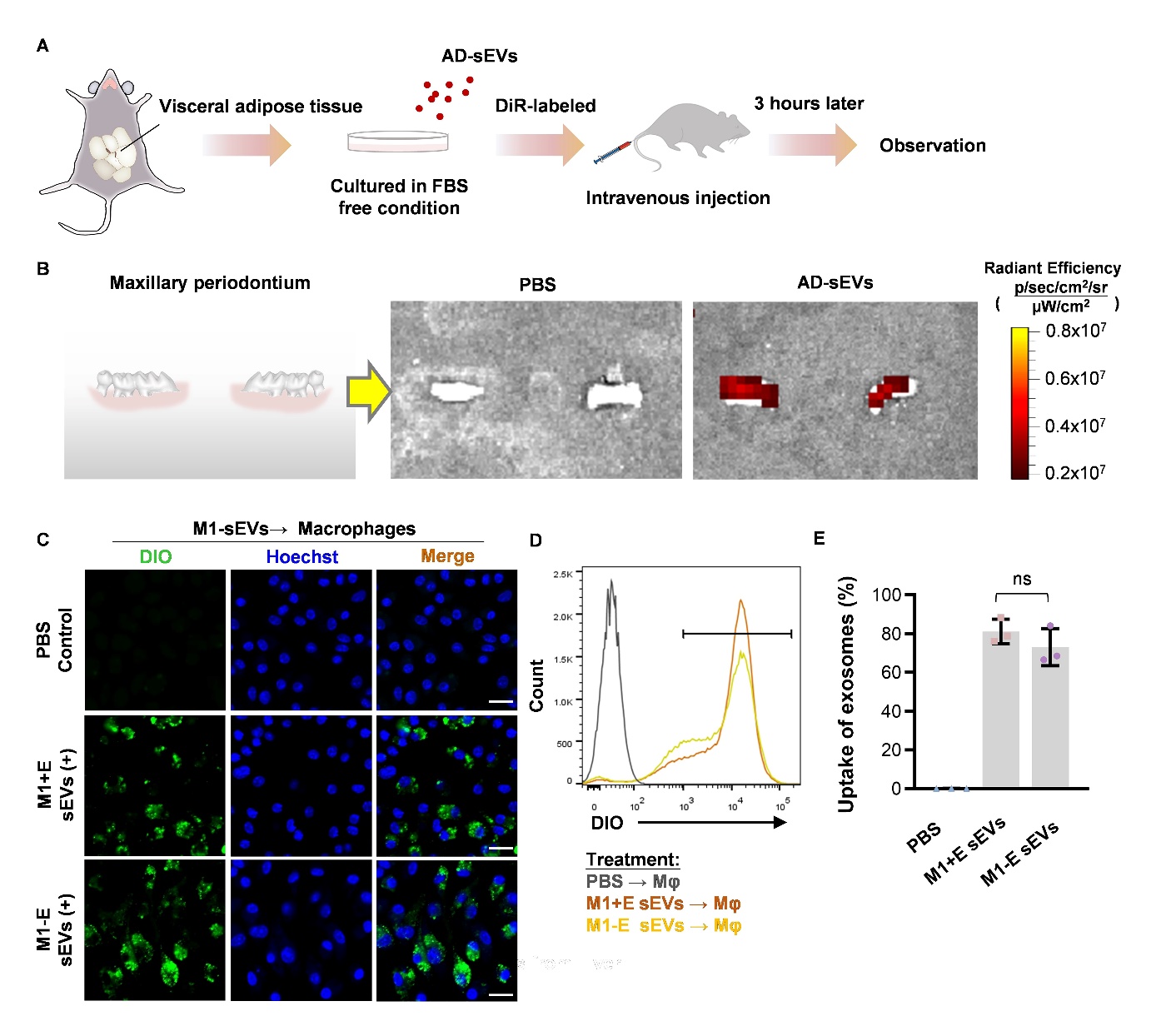
**

**Appendix Figure S2 -** **Fluorescent tracing experiments of macrophage derived small extracellular vesicles.**

A Illustration of injecting adipose tissue derived sEVs to mice and the tracing experiment.

B Luciferase activity at 3 hours later to examine the distribution in periodontal tissue of DIR-labeled-sEVs from adipose tissue (AD-sEVs) or an equal volume of PBS by in vivo imaging.

C Representative images of cell uptake of DiO labeled sEVs derived from M1 macrophages cultured in estrogen deficiency (M1-E sEVs, 5 μg) or in control condition (M1+E sEVs, 5 μg) by macrophages 6 hours after incubation (scale bar, 20 μm).

D-E Uptake of DIO labeled M1+E or M1-E sEVs by macrophages at 6 hours of incubation via flow cytometry. Data represent mean ± SEM. **P* < 0.05, ns: not significant. (One-way ANOVA).

**Appendix Table S1 - The methylation levels of every type C in the chromosome of Sham-BMDMs and OVX-BMDMs.**

**
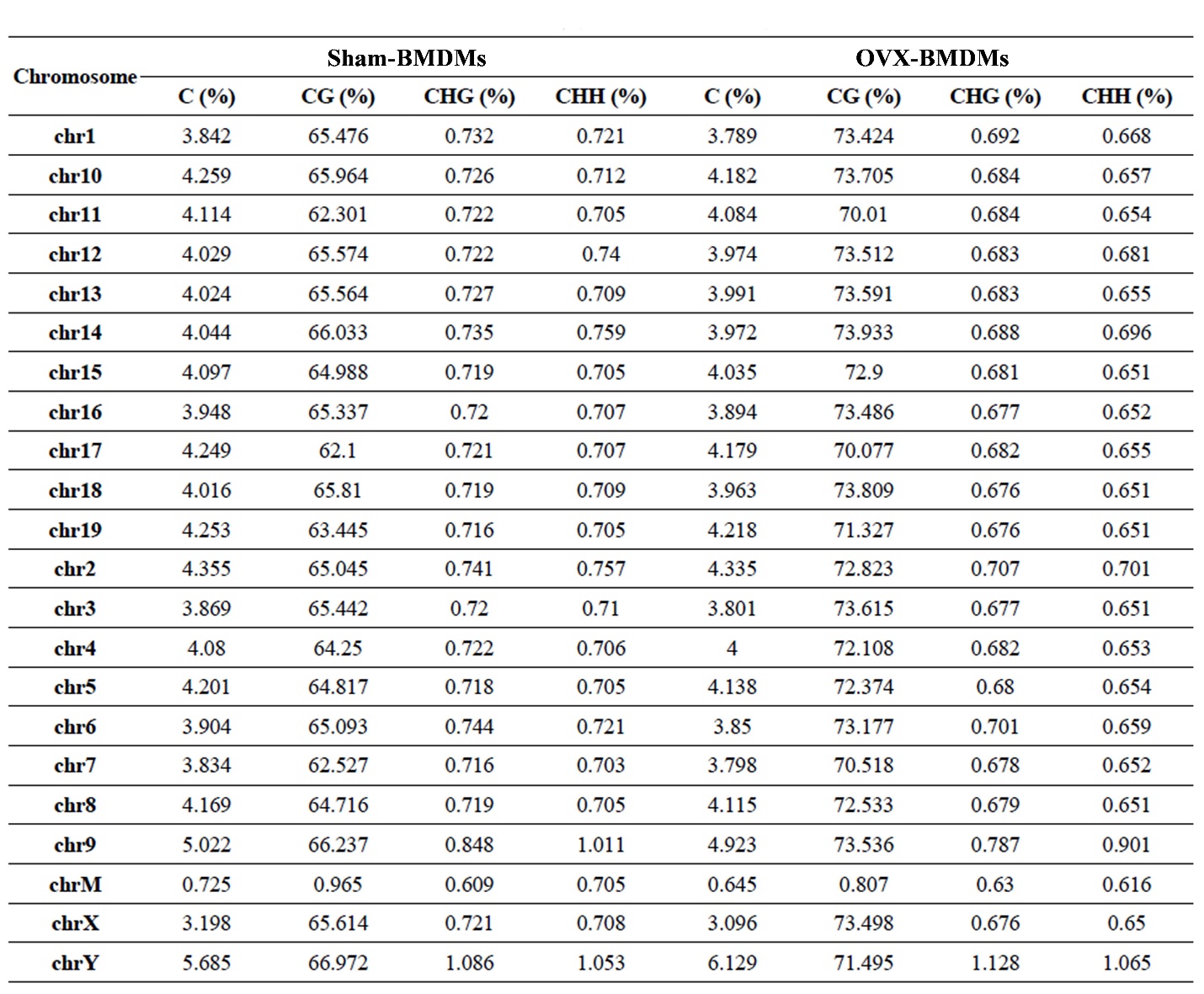
**

**Appendix Table S2 - Methylation levels of every type C in different functional elements in genome of Sham-BMDMs and OVX-BMDMs.**

**
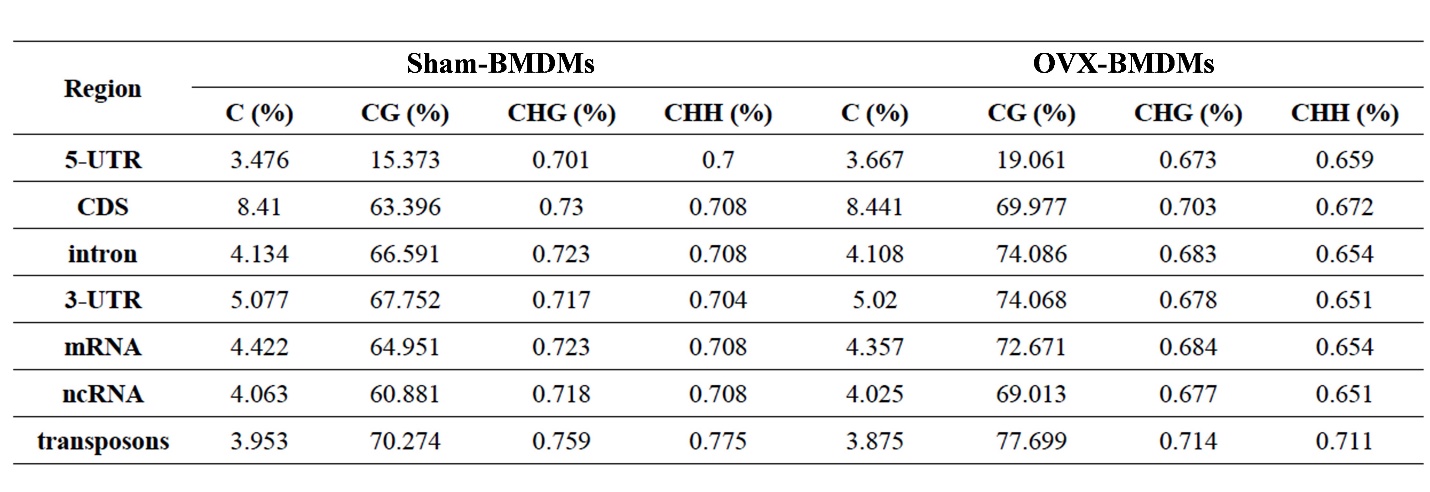
**

**Appendix Table S3 - The top 30 genes exhibiting the most significant differences in DNA methylation between Sham-BMDMs and OVX-BMDMs.**


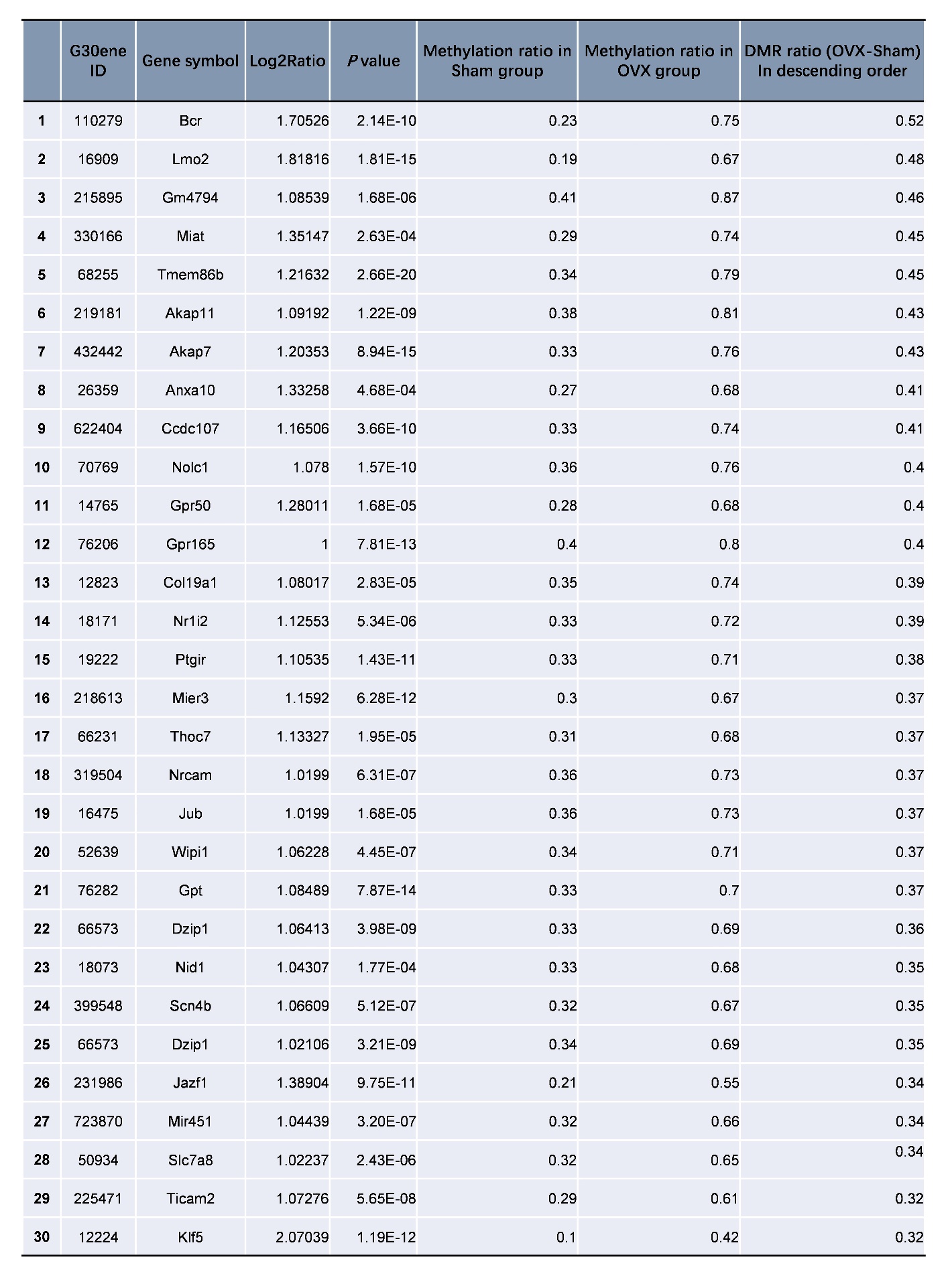


**Appendix Table S4 - Sequences of qPCR primers.**

| **Name** | **Sequence** | |
| --- | --- | --- |
| *β-actin* | Forward | 5’-GGCTGTATTCCCCTCCATCG-3’ |
|  | Reverse | 5’-CCAGTTGGTAACAATGCCATGT-3’ |
| *Gapdh* | Forward | 5’-AGGTCGGTGTGAACGGATTTG-3’ |
|  | Reverse | 5’-TGTAGACCATGTAGTTGAGGTCA-3’ |
| *Il1β* | Forward | 5’-GCAACTGTTCCTGAACTCAACT-3’ |
|  | Reverse | 5’-ATCTTTTGGGGTCCGTCAACT-3’ |
| *Il6* | Forward | 5’-TAGTCCTTCCTACCCCAATTTCC-3’ |
|  | Reverse | 5’-TTGGTCCTTAGCCACTCCTTC-3’ |
| *Tnfα* | Forward | 5’-CCCTCACACTCAGATCATCTTCT-3’ |
|  | Reverse | 5’-GCTACGACGTGGGCTACAG-3’ |
| *Nos2* | Forward | 5’-GTTCTCAGCCCAACAATACAAGA-3’ |
|  | Reverse | 5’-GTGGACGGGTCGATGTCAC-3’ |
| *Socs1* | Forward | 5’-CTGCGGCTTCTATTGGGGAC-3’ |
|  | Reverse | 5’-AAAAGGCAGTCGAAGGTCTCG-3’ |
| *Irf4* | Forward | 5’-AGGCAAAATCGGGAAGCACT-3’ |
|  | Reverse | 5’-CTGGGGGCTCGAACCATAC-3’ |
| *Jazf1* | Forward | 5’-GCCGAGAACAGGAATCTCTGA-3’ |
|  | Reverse | 5’-GTAAGGCTGCCACTGCTATGT-3’ |
| U6 | Forward | 5’-CTCGCTTCGGCAGCACA-3’ |
|  | Reverse | 5’-AACGCTTCACGAATTTGCGT-3’ |
| miR-30e-5p | Forward | 5’-GAAGGTCAGTTCCTACAAATGT-3’ |
| miR-125a-3p | Forward | 5’-ACAGGTGAGGTTCTTGGGAGCC-3’ |
| miR-155-5p | Forward | 5’-TTAATGCTAATTGTGATAGGGGT-3’ |

**Appendix Table S5 - Sequences of miRNA inhibitor and mimics.**

| **Name** | **Sequence** |
| --- | --- |
| miR-30e-5p inhibitor | 5’-CUUCCAGUCAAGGAUGUUUACA-3’ |
| microRNA inhibitor NC | 5’-CAGUACUUUUGUGUAGUACAA-3’ |
| miR-30e-5p mimics | Sense 5’-UGUAAACAUCCUUGACUGGAAG-3’  Anti-sense 5’-UCCAGUCAAGGAUGUUUACAUU-3’ |
| microRNA mimics NC | Sense 5’-UUCUCCGAACGUGUCACGUTT-3’  Anti-sense 5’-ACGUGACACGUUCGGAGAATT-3’ |
| Mus JAZF1-siRNA | Sense 5’-GGCCCUCAGUUACAUCAAUTT-3’  Anti-sense 5’-AUUGAUGUAACUGAGGGCCTT-3’ |
| NC-siRNA | Sense 5’-UUCUCCGAACGUGUCACGUTT-3’  Anti-sense 5’-ACGUGACACGUUCGGAGAATT-3’ |

**Appendix Table S6 - The sample size, average age, height, and weight of the enrolled subjects.**
